# Instrumented measures of sedentary behavior and physical activity are associated with depression among children and adolescents: a systematic review and dose–response meta-analysis of observational studies

**DOI:** 10.3389/fpsyg.2024.1465974

**Published:** 2024-10-04

**Authors:** Songtao Lu, Jun Sun, Zhiguang Guo, Mingyu Yi, Yuheng Zhang, Jiali Wang, Yue Wang

**Affiliations:** ^1^School of Physical Education, Central China Normal University, Wuhan, China; ^2^School of Sports, Wuhan University of Science and Technology, Wuhan, China; ^3^School of Sports Health, Hubei University of Chinese Medicine, Wuhan, China; ^4^Faculty of Artificial Intelligence in Education, Central China Normal University, Wuhan, China

**Keywords:** sedentary behavior, physical activity, accelerometry, depression, children and adolescents

## Abstract

**Background:**

Higher sedentary behavior (SB) and lower physical activity (PA) are associated with negative physical and mental health outcomes. SB and PA can be objectively assessed using inertial sensors to evaluate body movements. This study aimed to quantify the association between instrumented measures of SB (i-SB) and PA (i-PA) and depression among children and adolescents using a systematic review and meta-analysis of observational studies.

**Methods:**

An electronic search was conducted on six databases up to May 12, 2024. A dose–response meta-analysis was conducted to determine the association between i-SB and i-PA and depression, expressed as odds ratios (ORs) and 95% confidence intervals (CIs).

**Results:**

Five cross-sectional and 11 longitudinal studies comprising 26,109 participants met the inclusion criteria. Comparing the most sedentary with the least sedentary groups of participants resulted in a pooled ORs of 1.05 (95% CI 0.94–1.16). Comparing the least active with the most active groups of participants resulted in pooled ORs of 0.93 (95% CI 0.84–1.07), 0.89 (95% CI 0.79–1.00), 0.83 (95% CI 0.66–0.99), and 0.73 (95% CI 0.58–0.89) for light, moderate-to-vigorous (MV), vigorous, and total PA, respectively. Robust error meta-regression analyses showed clear dose–response relationships between i-SB and i-MVPA and depression.

**Conclusion:**

Both i-SB and i-PA were significantly associated with risk of depression in children and adolescents, which may become non-significant after mutual adjustment for i-PA and i-SB.

**Systematic review registration:**

[https://www.crd.york.ac.uk/PROSPERO/display_record.php?RecordID=546666], identifier [CRD42024546666].

## 1 Introduction

Adolescents and children undergo many physical and psychological changes, including the development of reproductive organs, the formation of sexual characteristics, and the improvement of cognitive abilities. Although individuals reach physical maturity in early adulthood, their psychological, social, and emotional aspects are still developing. Adolescence and early adulthood are critical transitional periods from childhood to adulthood, and their health status during these periods lays the foundation for long-term health and life (Sawyer et al., 2012). Anxiety and depression are the most common mental disorders that begin in adolescence and early adulthood (McGrath et al., 2023) and have become serious public health problems. Depression is a common mental health problem among adolescents and children that often manifests as reduced motivation, low learning efficiency, difficulty in interpersonal communication, disordered eating and sleep, and in severe cases, even the intention or behavior of self-harm or suicide (Park et al., 2019). In recent decades, the global prevalence of depressive symptoms among adolescents has increased from 24 to 37% (Shorey et al., 2022), and the prevalence among adults has shown a similar rapid growth trend (World Health Organization [WHO], 2017).

Increasing evidence suggests that physical activity (PA) can reduce symptoms of depression in both clinical and non-clinical populations (Kandola et al., 2019). Most previous studies have focused on moderate to vigorous intensity (MVPA), such as brisk walking or cycling, which appears to modulate several biological and psychosocial pathways to reduce depressive symptoms. In 2010, the World Health Organization (WHO) recommended that children aged 5–17 years engage in at least 1 h of MVPA per day (World Health Organization [WHO], 2024). Evidence also suggests that the time spent in sedentary behavior (SB) is associated with the risk of depression (Teychenne et al., 2010). Moreover, sitting behavior, independent of PA levels, is a risk factor for cardiovascular metabolic diseases, various causes of death, and physiological and psychological problems (Katzmarzyk et al., 2009). Screen-based behavior (including watching TV, DVDs, and videos and using computers, smartphones, and iPads) is the most common form of SB in children and should not exceed 2 h per day (Colley et al., 2011). Although some progress has been made in understanding the relationship between PA, SB, and the physical and mental health of children and adolescents, more in-depth research is needed to understand the complex relationships involved fully.

The association between PA and depression has also been previously quantified in a meta-analysis, resulting in an odds ratio (OR) of up to 0.90 (95% confidence interval [CI] 0.84–0.98) for an active adolescent compared to an active adolescent with a relatively high level of PA (Schuch et al., 2018). The association between SB and depression in childhood and adolescents has been meta-analyzed and quantified previously, resulting in a pooled relative risk of 1.14 (95% CI 1.08–1.20) (Zhang et al., 2022) and 1.12 (CI 1.03–1.22) (Liu et al., 2016). Most studies included in these meta-analyses used self-reported PA measures. However, self-reported measures tend to overestimate PA and underestimate SB (Chinapaw et al., 2009; Ryan et al., 2018). A previous study reported that 76% of young-to-middle-aged adults (*n* = 119) and 90% of older adults (*n* = 72) met the recommended Dutch PA guidelines, although instrumented measures of PA (i-PA) indicated that only 24% (*n* = 37) and 16% (*n* = 13) of participants objectively met these criteria (Rojer et al., 2018). Therefore, it is important to quantify the association between i-SB and i-PA and depression and to compare effect sizes for the development of lifestyle-related guidelines in childhood and adolescence.

Although a previous meta-analysis assessed the association between objectively measured i-PA levels and the incidence and prevalence of depression in adults, no meta-analysis has reported the association in childhood or adolescence. Furthermore, there has never been a meta-analysis on the association between objectively measured i-SB and depression. Recent evidence from a cohort study has shown an important joint role of daily PA and SB in depression risk in adolescent populations (Monteagudo et al., 2023). Therefore, this systematic review and meta-analysis aimed to quantify the association between i-SB and i-PA and depression in childhood and adolescence, compare the quantitative effect sizes of i-SB and i-PA, and determine the dose–response relationship between i-SB and i-PA and depression risk.

## 2 Methods

### 2.1 Search strategy

The study protocol for this systematic review was registered in the PROSPERO International Prospective Register of Systematic Reviews (registration number CRD42024546666). Following the PRISMA guidelines, researchers systematically searched electronic databases for literature on the association between PA and depression. The systematic search included the PubMed, EMBASE.com, Cochrane Library (via Wiley), Scopus, PsycINFO, and SPORTDiscus (via EBSCO) bibliographic databases up to May 12, 2024. We searched for articles using the search terms “depression OR depressive symptoms; physical activity OR exercise OR physical training; sedentary behavior OR behavior; sedentary OR sedentary behaviors; sedentary lifestyle OR lifestyle; sedentary OR physical sedentary OR physical inactivity; adolescents OR adolescent OR teens OR child OR children; accelerometry OR accelerometer OR pedometer OR monitoring” with a series of prospective studies. In addition, we searched the reference lists of all the included studies to identify additional eligible articles. No search language restrictions were applied. The full search strategy is presented in Supplementary Tables S1–S6.

### 2.2 Study selection

Two of the three reviewers (YW and ZG) independently screened the titles and abstracts, followed by the full texts of all studies for eligibility according to the inclusion and exclusion criteria. Discrepancies were resolved by adjudication by a third independent reviewer (ST). The articles were organized and managed using EndNote (Version X8.2. Clarivate Analytics, Philadelphia, PA, United States). Articles published in full text in English or Chinese were considered eligible if they (1) were observational cohort, cross-sectional, and case–control studies that assessed SB or PA using pedometry or accelerometry; (2) included depression, depressive symptoms, or major depression as an outcome measure; (3) included children and adolescents no older than 20 years; (4) focused on childhood and adolescence without selection of a specific disease group; and (5) reported ORs (odds ratios), RR (relative risks), HR (hazard ratios), and 95% CIs of PA and SB related to the risk of depression (or provided raw data to calculate these indicators). If multiple articles were published on the same population, those with longer tracking times or larger sample sizes were included in the analysis. The researchers searched the literature separately according to the above criteria, identified studies that met the requirements and discussed the studies to determine whether they should be included. Studies that purposefully selected participants with specific diseases were excluded from analysis. Studies conducted in controlled environments such as exercise laboratories or review articles were excluded.

### 2.3 Statistical analyses

Meta-analysis was performed using STATA software (version 16.0), and all tests were two-sided. We performed a two-step meta-analysis of categorical and continuous variables to assess the association between PA and the risk of depression. Pooled results are expressed as ORs with 95% CIs. Pooled analyses were categorized into cohort and cross-sectional studies based on the study type, and pooled ORs were estimated using a random-effects model. Heterogeneity was assessed using the I^2^ statistic as the percentage of variation in the study, with *I*^2^ values of 25, 50, and 75% indicating low, medium, and high heterogeneity, respectively (Higgins et al., 2019). The *p*-value for assessing heterogeneity was set at 0.05. Egger and Begg’s tests were used to determine whether publication bias existed (Higgins et al., 2019). For the sensitivity analysis, each study was removed individually to check whether the combined effect of the remaining studies had changed. Subgroup meta-analysis was conducted by study type, sex, age, study area, study quality, and adjustment for confounding factors. Meta-regression was used to examine heterogeneity among studies.

To analyze the continuous dose–response relationship, we calculated the total weekly duration of PA and SB for each OR provided in the literature. We assumed that the durations of PA and SB remained at this level during the follow-up survey. The median was set as the determined dose to determine the exposure values of the included doses. If the development interval was <0.5, it was set as 0.25. If the upper open interval was ≥1, the difference between the intermediate dose intervals was 0.25; therefore, the exposure value was 1.25 (Zhang et al., 2015).

A robust error meta-regression method described by Xu and Doi et al. (2018) was used to calculate continuous dose–response relationship slopes (non-linear trends) and 95% CIs from the natural logs of the reported ORs and CIs across the categories of PA and SB measures. This method is based on a one-stage approach that considers each study as a cluster of the entire sample and treats within-study correlations using clustered robust errors. Based on the goodness-of-fit test of the model, the Stata software XBLC command was used to draw a dose–response curve (Zhang et al., 2015).

### 2.4 Quality assessment

The Newcastle-Ottawa Scale (NOS) was used to assess the quality of the literature. The higher the overall score, the higher the study quality, with a maximum score of 9. Studies with NOS scores of 0–3, 4–6, and 7–9 were considered low, moderate, and high quality, respectively (Wells et al., 2009). If there was an inconsistent evaluation of the quality of the literature, the group focused on this issue and determined a final score based on quality.

## 3 Results

### 3.1 Description of included studies

**Figure 1:**
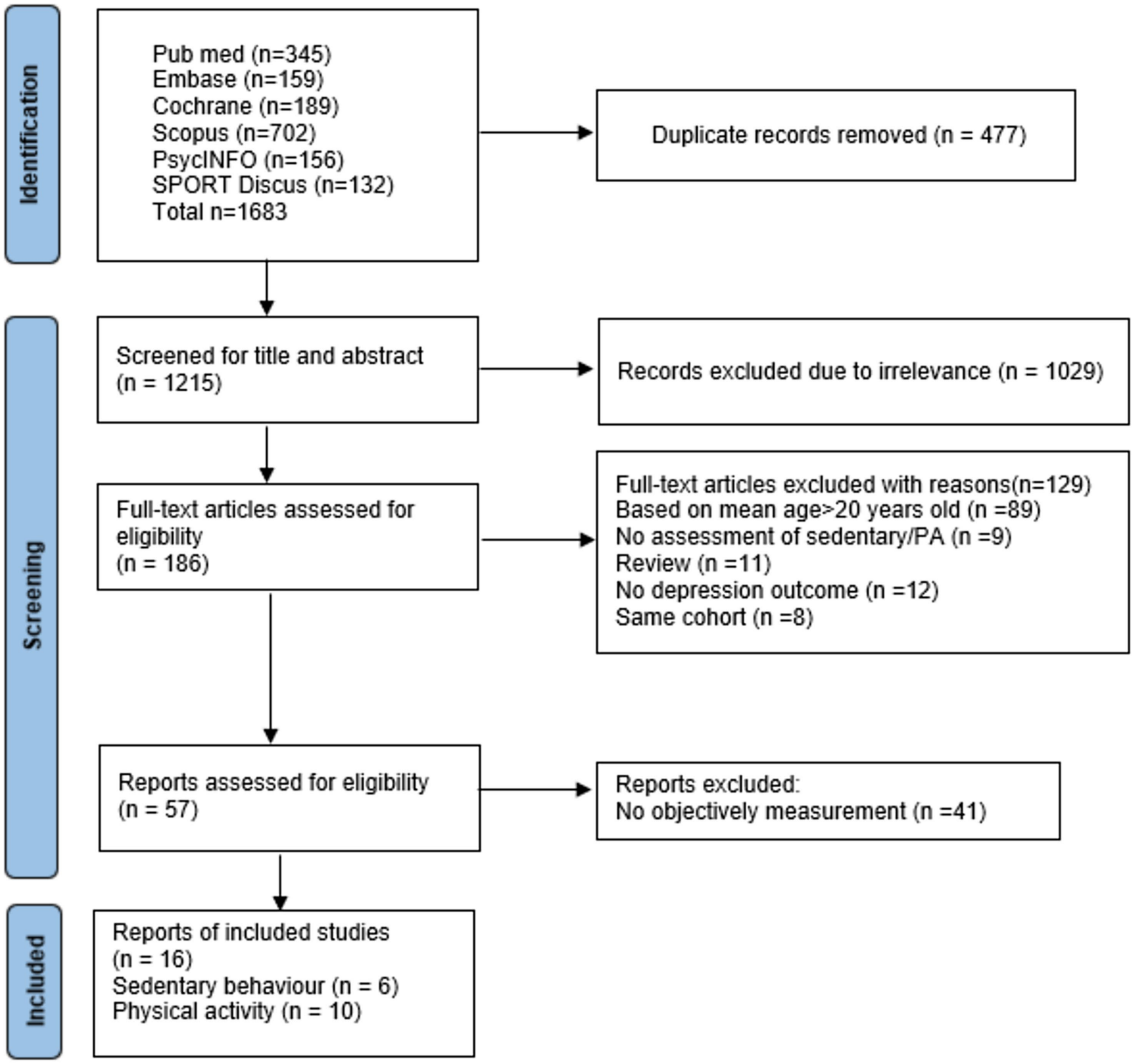
PRISMA flow chart of included studies.

We initially identified 1,683 articles, of which 1,215 articles remained after duplicates were removed. After screening titles and abstracts, 186 full-text articles were assessed for eligibility. One article was added after checking references in the final analysis, resulting in 16 articles in the final analysis comprising 11 cohort studies (Kracht et al., 2023; Booth et al., 2023; Hamer et al., 2020; Kandola et al., 2020; Slykerman et al., 2020; Toseeb et al., 2014; Hagemann et al., 2021; Wiles et al., 2011; McKercher et al., 2014; Olive et al., 2016; Bell et al., 2019) and five cross-sectional studies (Cushing et al., 2018; Farren et al., 2018; da Costa et al., 2022; Hrafnkelsdottir et al., 2018; Hume et al., 2011). A visualization of the article selection process is shown in Figure 1.

Table 1 shows the characteristics of the 16 included studies that included 26,109 participants from 11 cohorts and 5 cross-sectional studies conducted in Europe (*n* = 11), the United States (*n* = 3), and Oceania (*n* = 2). The participants’ mean age ranged from 12.0 to 15.8 years, and 39.0–63% of the participants were female. Depression rates varied from 3.2 to 48.8% among studies (Supplementary Table S7). The methodological quality of the included studies is presented in Supplementary Table S8. In total, 13 of 16 studies were reported as high quality. Only one study was reported to be of low quality (Supplementary Table S9).

**Table 1:** Characteristics of included studies.

Autho r(year)	Country	Study name or population	Age (M, SD or range)	No. of participants	PA measures	Wearing location	FU (years)	Quality Evaluation
Kracht et al. (2023)	USA	Adolescents aged 10–16 years	12.5 (2)	205 (females = 54.6%)	SB; MVPA	Midaxillary line	2	9
Booth et al. (2023)	UK	ALSPAC	11 ~ 13	4,755 (females = 54.2%)	MVPA; TPA	The right hip	N/A	8
Hamer et al. (2020)	UK	Children in the UK	7 ~ 14	6,675 (females = 53.5%)	MVPA; LPA; SB	Waist	7	7
Kandola et al. (2020)	UK	ALSPAC	17 ~ 18	4,257 (females = 56.1%)	SB; LAP; MVPA	The right hip	6	7
Slykerman et al. (2020)	NZ	ABC	11 ~ 16	871 (females = 50.7%)	SB; MVPA; VPA	N/R	16	6
Toseeb et al. (2014)	UK	ROOTS	15 (3.6)	736 (females = 56.8%)	PAEE; MVPA	Left chest	3	8
Hagemann et al. (2021)	BE	SIGMA	11 ~ 19	934 (females = 63%)	LPA; MPA; VPA	Wrist	N/A	5
Wiles et al. (2011)	UK	The ALSPAC study	13.8 (0.21)	3,298 (females = 53%)	TPA; MVPA	Waist, right hip	N/A	7
McKercher et al. (2014)	AUS	CDAHS	9 ~ 15	1,630 (females = 53.4%)	Steps; MVPA	N/R	20	9
Olive et al. (2016)	AUS	Grade 2 children aged 7 to 9 years	8.18–8.13	791 (females = 49.8%)	Steps; LPA; MVPA	Hip	8	9
Bell et al. (2019)	UK	Students in West England	12 ~ 13	673 (females = 51%)	SB; LPA; MPA; VPA	N/R	3	6
Hume et al. (2011)	AUS	CLANS	14.4 (0.61)	155 (females = 60%)	MVPA; VPA; SB	N/R	2	8
Cushing et al. (2018)	USA	College student	15.67 (1.56)	26 (females = 42.3%)	SB; LAP; MVPA	Non-dominant wrist	20	8
da Costa et al. (2022)	BR	Brazilian adolescents	14 ~ 18	610 (females = 51.6%)	MVPA; LPA; SB	Non-dominant wrist	4	10
Farren et al. (2018)	USA	Middle school student in Texas	12.85 (0.89)	249 (female = 54%)	MVPA	Wrist	N/A	10
Hrafnkelsdottir et al. (2018)	IS	10th graders in Iceland	15.8	244 (females = 59%)	VPA; TPA; screen time	Non-dominant wrist	N/A	10

FU, follow up; M, mean age; SD, standard deviation; IQR, interquartile range; N/A, not applicable; N/R, not reported; Q, quartile; T, tertile; S, subgroup; M, in male; F, in female; CAN, Canada; USA, United States of America; UK, United Kingdom; AUS, Australia; NZ, New Zealand; IS, Iceland; BR, Brazil; BE, Belgium; ABC Study, the Auckland Birthweight Collaborative Study; ALSPAC, the Avon Longitudinal Study of Parents and Children cohort; CDAHS, The Childhood Determinants of Adult Health Study; SIGMA, Slim Initiative in Genomic Medicine for the Americas; ROOTS, Risk, Obesity, and Other Traits in Schoolchildren. CLANS, Children Living in Active Neighborhoods study.

### 3.2 Measurement method

Supplementary Table S8 shows an overview of the measurement devices used, including ActiGraph (Kracht et al., 2023; Booth et al., 2023; Hamer et al., 2020; Kandola et al., 2020; Slykerman et al., 2020; Toseeb et al., 2014; Wiles et al., 2011; Olive et al., 2016; Cushing et al., 2018; Farren et al., 2018; Hume et al., 2011; Hrafnkelsdottir et al., 2018), Actiheart (da Costa et al., 2022), Fitbit Charge (Hagemann et al., 2021), Yamax Digiwalker (McKercher et al., 2014), and Philips Electronics (Bell et al., 2019). Five different types of accelerometers, two different pedometers, and three other devices, including hip, wrist, thigh, and triceps-worn devices, were used. Furthermore, multiple cut-off values were used to assess i-SB and i-PA. Measurements of at least four consecutive days were required (Van Schooten et al., 2015). Most of the included studies had a measurement period of seven consecutive days.

### 3.3 Results for i-SB and i-PA

#### 3.3.1 SB and depression

**Figure 2:**
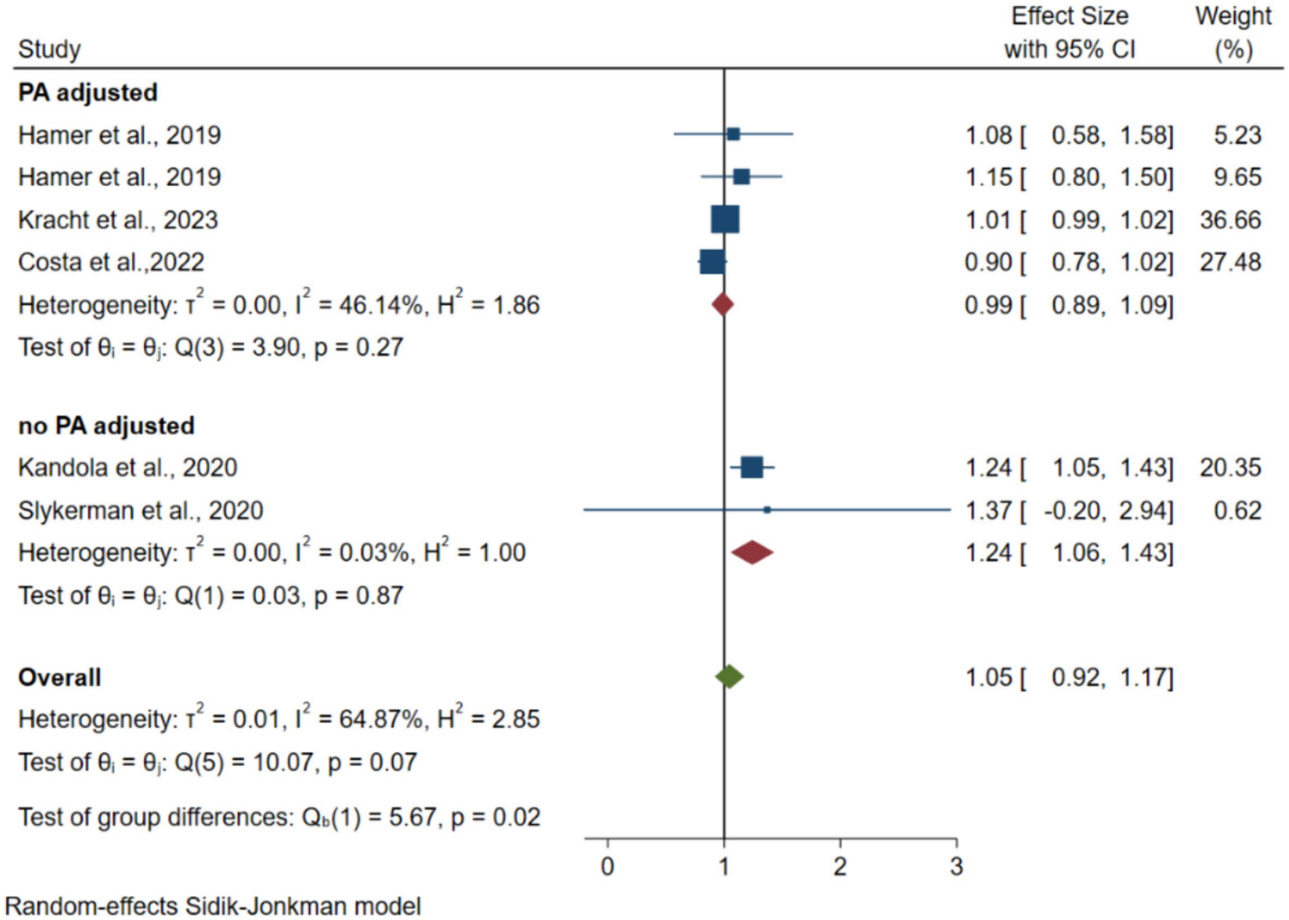
Forest plots of the association between instrumented SB and depression.

Five articles reporting 12,618 participants were included (Kracht et al., 2023; Hamer et al., 2020; Kandola et al., 2020; Slykerman et al., 2020; da Costa et al., 2022). Figure 2 shows forest plots of the association between SB and depression risk. The pooled random-effects model resulted in an OR of 1.05 (95% CI 0.92–1.17, *p* = 0.38 > 0.05, *I*^2^ = 64.9). Four data points from three articles adjusted for time spent in PA after the pooled random-effects model resulted in an OR of 0.99 (95% CI 0.89–1.09, *p* = 0.91 > 0.05, *I*^2^ = 46.1). Data from two articles with no adjusted PA resulted in an OR of 1.24 (95% CI 1.06–1.43, *p* = 0.003 < 0.05, *I*^2^ = 0). The test of group difference (*p* = 0.02) showed a significant difference between PA adjusted and no PA adjusted for OR value. The sensitivity analysis revealed no significant changes in the combined effect measures when each study was excluded.

#### 3.3.2 Light PA and depression

**Figure 3:**
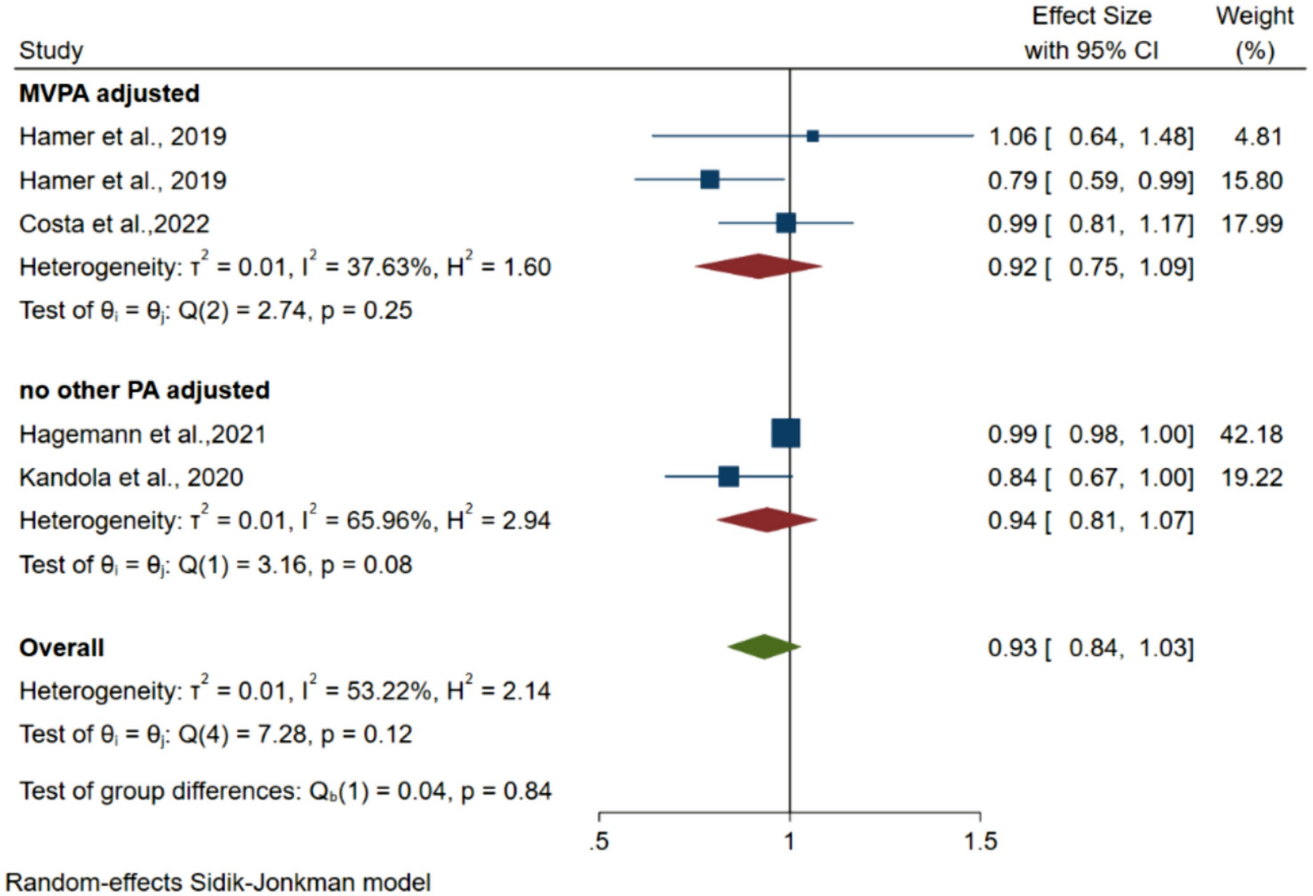
Forest plots of the association between instrumented LPA and depression.

Four articles with 12,476 participants reported an association between light PA (LPA) and depression (da Costa et al., 2022; Kandola et al., 2020; Hamer et al., 2020; Hagemann et al., 2021). Figure 3 shows forest plots of the association between LPA and depression risk. The pooled random-effects model resulted in an OR of 0.93 (95% CI 0.84–1.07, *p* = 0.37 > 0.05, *I*^2^ = 53.2), 0.92 (95% CI 0.75–1.09, *p* = 0.42 > 0.05, *I*^2^ = 37.6), 0.94 (95% CI 0.81–1.07, *p* = 0.24 > 0.05, *I*^2^ = 65.9) for overall, MVPA adjusted, no other PA adjusted. All the pooled OR values were not statistically significant.

#### 3.3.3 MVPA and depression

**Figure 4:**
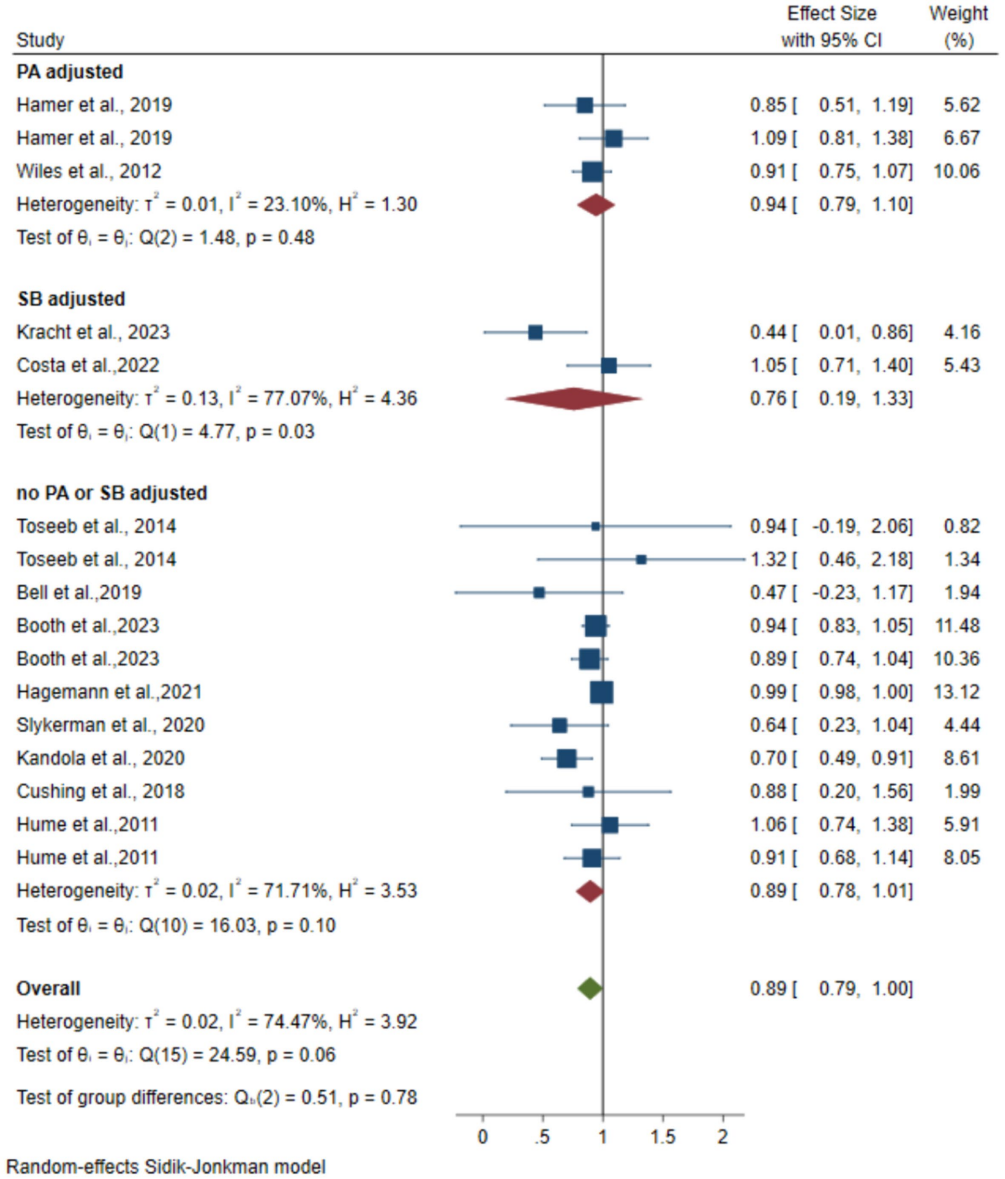
Forest plots of the association between instrumented MVPA and depression.

Twelve articles with 23,195 participants (Kracht et al., 2023; Booth et al., 2023; Hume et al., 2011; Hamer et al., 2020; Slykerman et al., 2020; Kandola et al., 2020; Toseeb et al., 2014; Hagemann et al., 2021; Wiles et al., 2011; Bell et al., 2019 Cushing et al., 2018; da Costa et al., 2022) reported an association between MVPA and the risk of depression. Figure 4 shows forest plots of the association between MVPA and depression risk. The pooled random-effects model resulted in an OR of 0.89 (95% CI 0.79–1.00, *p* = 0.02 < 0.05, *I*^2^ = 74.47) and 0.89 (95% CI 0.78–1.01, *p* = 0.03 < 0.05, *I*^2^ = 71.71) for the overall and no PA or SB adjusted groups. After adjustment of two data for SB, the pooled random-effects model resulted in an OR of 0.76 (95% CI 0.19–1.33, *p* = 0.87 > 0.05, *I*^2^ = 0). After adjustment of three data points for lower levels of PA, the pooled random-effects model resulted in an OR of 0.94 (95% CI 0.79–1.10, *p* = 0.43 > 0.05, *I*^2^ = 0). The combined effect quantity did not exhibit significant changes when each study was excluded from the sensitivity analysis.

#### 3.3.4 Vigorous PA and depression

**Figure 5:**
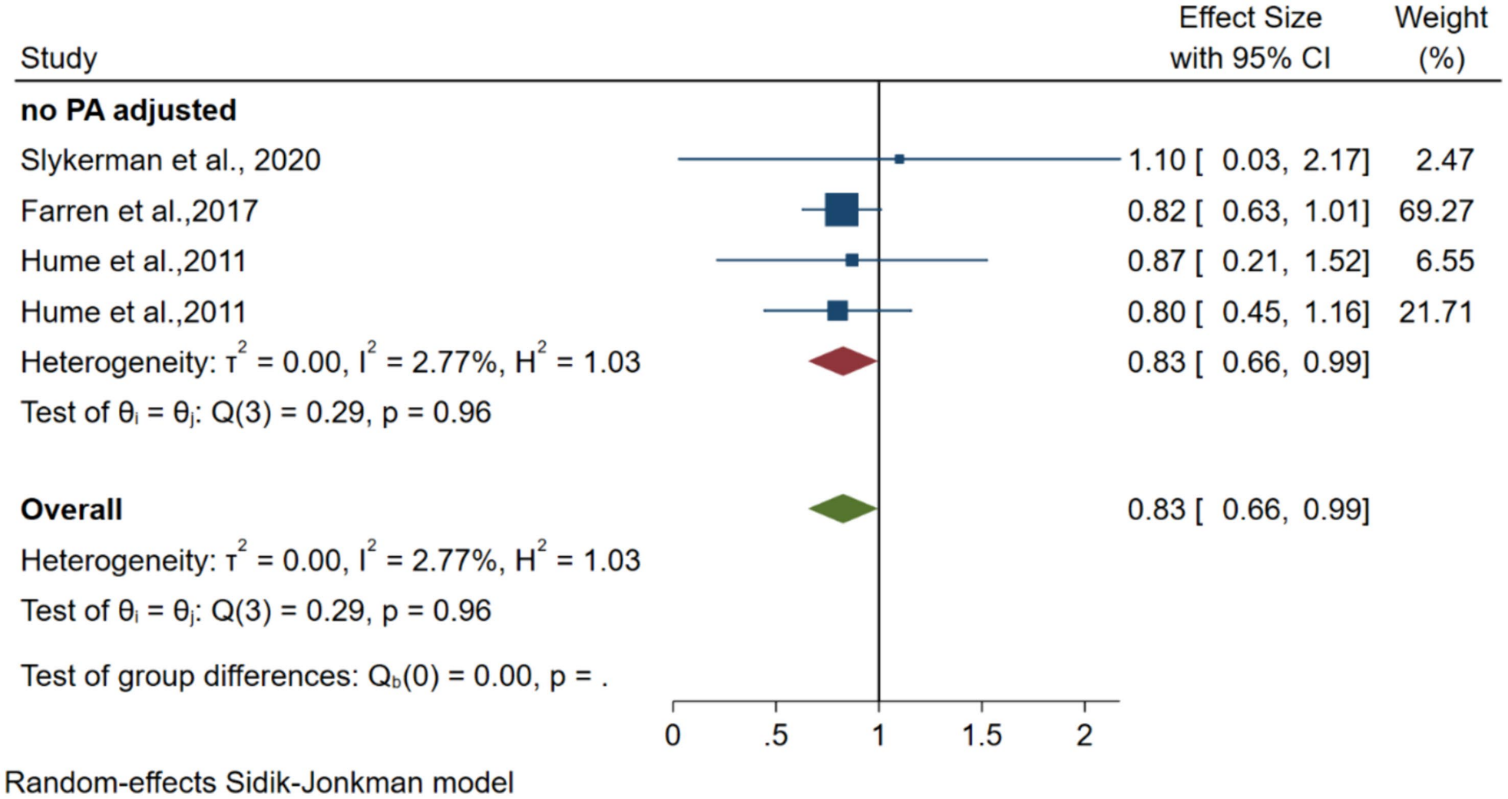
Forest plots of the association between instrumented MVPA and depression.

Three articles with 1,275 participants (Slykerman et al., 2020; Hume et al., 2011; Farren et al., 2018) reporting on the association between vigorous PA and depression risk were included. Figure 5 shows forest plots of the association between vigorous PA and the risk of depression. The pooled random-effects model resulted in an OR of 0.83 (95% CI 0.66–0.99, *p* = 0.07 > 0.05, *I*^2^ = 2.77).

#### 3.3.5 Total PA and depression

**Figure 6:**
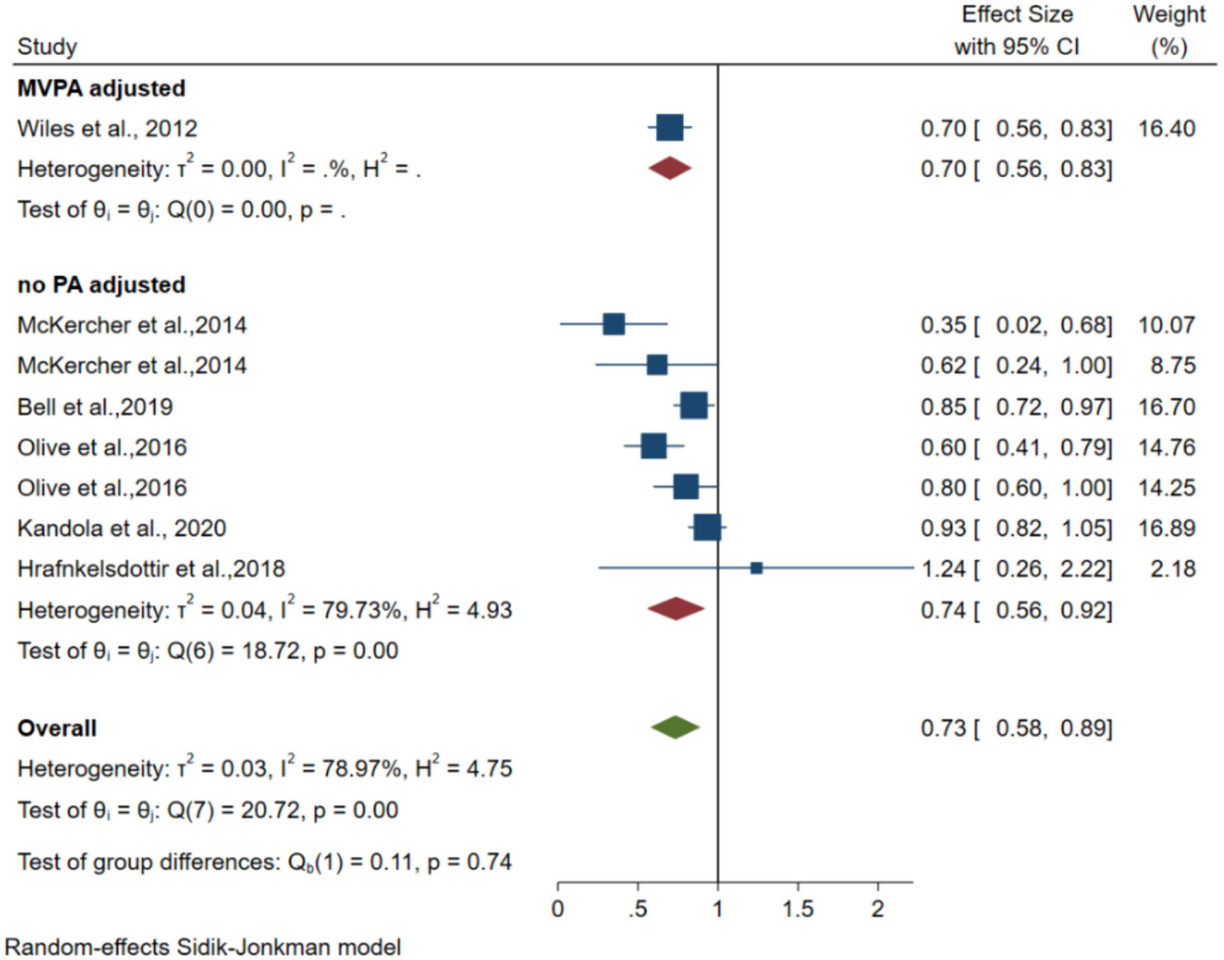
Forest plots of the association between instrumented TPA and depression.

Six articles including 10,893 participants (Kandola et al., 2020; Wiles et al., 2011; McKercher et al., 2014; Bell et al., 2019; Olive et al., 2016; Hrafnkelsdottir et al., 2018) reported an association between TPA and depression risk. The pooled random-effects model comparing the relatively inactive with the relatively active group resulted in an OR of 0.73 (95% CI 0.58–0.89, *p* = 0.00 < 0.05, *I*^2^ = 78.9). After adjusting one data point for MVPA, the pooled random-effects model resulted in an OR of 0.70 (95% CI 0.58–0.83). The forest plot is shown in Figure 6.

### 3.4 Subgroup analysis

Table 2 shows the results of the subgroup analyses examining the stability of the pooled ORs and exploring the potential sources of heterogeneity for MVPA and SB. In general, the association between MVPA and depression was consistent in most of the between-group analyses. Regarding the source of within-subgroups analysis, significant heterogeneity was found in the cohort studies, mixed sex, depression scales (others), adjusted for sex, unadjusted BMI, and baseline disease confounding factors, indicating that these factors may affect the pooled ORs.

**Table 2:** Results of the subgroup analysis by meta-regression.

Subgroup		MVPA					SB				
		*N*	OR (95% CI)	*I*^2^ (%)	*p* ^a^	*P* ^b^	*N*	OR (95% CI)	*I*^2^ (%)	*p* ^a^	*P* ^b^
All reports		16	0.89 (0.79,1.00)	74.47	0.06	–	6	1.05 (0.92,1.17)	64.87	0.07	–
Study design	Cohort	12	0.86 (0.74,0.99)	81.41	0.01	**0.06**	5	1.08 (0.97,1.20)	31.60	0.15	**0.03**
	CS	4	0.98 (0.81,1.14)	4.40	0.84		1	0.90 (0.78,1.20)	–	–	
Research quality	≥7	14	0.89 (0.78,1.01)	58.49	0.35	0.94	5	1.04 (0.92,1.17)	69.80	0.04	0.69
	7<	2	0.88 (0.57,1,19)	62.81	0.09		1	1.37(−0.20,2.94)	–	–	
Gender	Male	3	0.95 (0.82,1.07)	12.71	0.67	0.54	1	1.08 (0.58,1.58)	–	–	0.85
	Female	3	0.94 (0.80,1.08)	24.77	0.47		1	1.15 (0.80,1.50)	–	–	
	Mixed	10	0.83 (0.67,1.00)	71.38	0.02		4	1.04 (0.87,1.21)	82.23	0.02	
Age	<14	8	0.89 (0.75,1.04)	85.47	0.07	0.94	3	1.02 (0.95,1.08)	4.19	0.70	0.71
	>14	8	0.88 (0.72,1.05)	34.52	0.40		3	1.06 (0.81,1.32)	68.86	0.01	
Depression scales	CES-D	4	0.93 (0.73,1.13)	39.07	0.38	0.82	2	0.91 (0.64,1.19)	4.76	0.56	0.48
	SDQ	5	0.92 (0.75,1.08)	3.41	0.49		2	1.13 (0.84,1.41)	0.12	0.82	
	Other	7	0.85 (0.66,1.04)	74.58	0.02		2	1.10 (0.89,1.32)	81.50	0.02	
Region	Europe	11	0.90 (0.78,1.0)	75.79	0.07	0.71	4	1.21 (1.04,1.37)	3.42	0.92	**0.02**
	America	3	0.79 (0.41,1.18)	52.31	0.09		2	0.97 (0.87,1.07)	66.11	0.07	
	Oceania	2	0.96 (0.76,1.16)	10.81	0.46		0	–	–	–	
Wearing location	Wrist	3	0.99 (0.95,1.03)	1.89	0.90	0.11	1	0.90 (0.78,1.02)	–	–	**0.01**
	Hip	6	0.90 (0.79,1.00)	47.65	0.33		3	1.20 (1.04,1.37)	66.11	0.07	
	Others	7	0.81 (0.56,1.05)	51.18	0.18		2	1.01 (0.86,1.16)	1.82	0.65	
Confounding factor											
Age	Yes	7	0.89 (0.73,1.05)	87.83	0.06	0.96	3	0.97 (0.84,1.10)	67.23	0.18	**0.03**
	No	9	0.89 (0.74,1.04)	34.89	0.38		3	1.20 (1.04,1.37)	2.99	0.79	
Sex	Yes	12	0.89 (0.77,1.02)	80.96	0.03	0.97	4	1.08 (0.97,1.20)	37.63	0.09	0.26
	No	4	0.90 (0.69,1.10)	26.10	0.47		2	0.91 (0.64,1.19)	4.76	0.56	
BMI	Yes	11	0.93 (0.81,1.05)	56.12	0.55	0.33	4	0.99 (0.89,1.09)	46.14	0.27	**0.02**
	No	5	0.82 (0.63,1.00)	55.47	0.01		2	1.24 (1.06,1.43)	0.03	0.87	
SES	Yes	13	0.89 (0.77,1.01)	62.89	0.28	0.86	5	1.04 (0.92,1.17)	69.80	0.04	0.69
	No	3	0.91 (0.71,1.12)	32.64	0.23		1	1.37 (−0.20,2.94)	–	–	
Ethnicity	Yes	8	0.84 (0.70,0.99)	71.41	0.11	0.24	4	1.08 (0.97,1.20)	37.63	0.09	0.26
	No	8	0.96 (0.83,1.10)	36.17	0.74		2	0.91 (0.64,1.19)	4.76	0.56	
Baseline disease	Yes	7	0.93 (0.78,1.09)	61.54	0.67	0.50	0	–	–	–	–
	No	9	0.86 (0.72,1.00)	64.34	0.02		6	1.05 (0.92,1.17)	64.87	0.07	

CS, cross-section; SES, socioeconomic status; *P*^a^ and *P*^b^, heterogeneity within and between subgroups, respectively; wearing location, wearing location of the instrumented measures. The bold values means significant.

As for SB, Sources of significant between-group heterogeneity among included studies may be found in the study region, wearing location, region, and adjustment variables. Regarding the source of within-subgroups analysis, significant heterogeneity was found in the research quality (≥7), mixed sex, age (>14), depression scales (others), adjusted for SES, indicating that these factors may affect the pooled ORs.

### 3.5 Publication bias

Funnel plots were constructed for all studies included in the SB meta-analysis. The funnel plot showed no asymmetry (Supplementary Figure S1A). Egger’s regression test (one-tailed *p* = 0.29) indicated no statistically significant publication bias. However, the funnel plot showed evidence of asymmetry in MVPA levels (Supplementary Figure S1B). Egger’s regression test (one-tailed, *p* = 0.004) indicated a statistically significant publication bias.

### 3.6 Dose–response relationship

**Figure 7:**
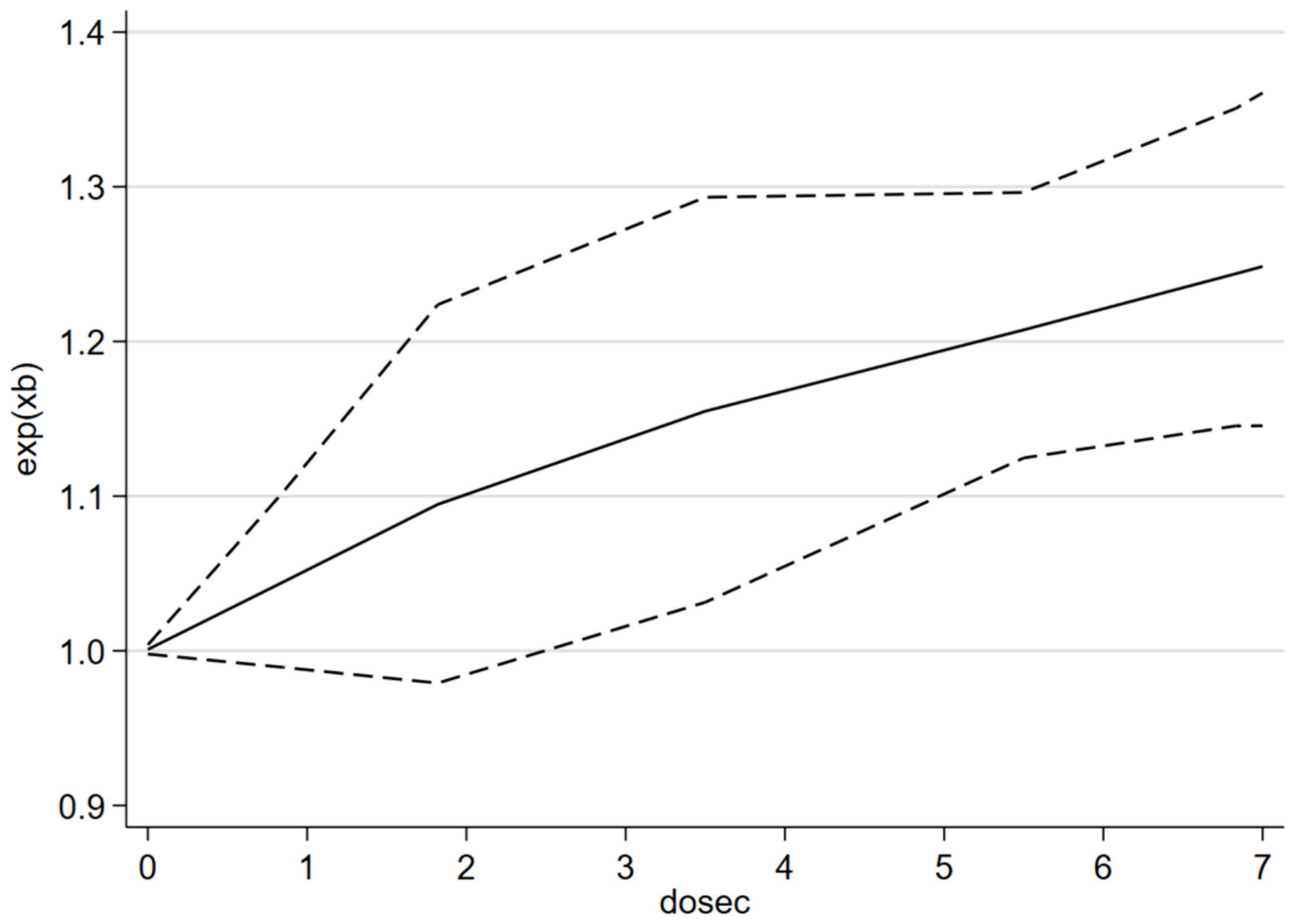
The dose–response relationship between i-SB and the risk of depression.

**Figure 8:**
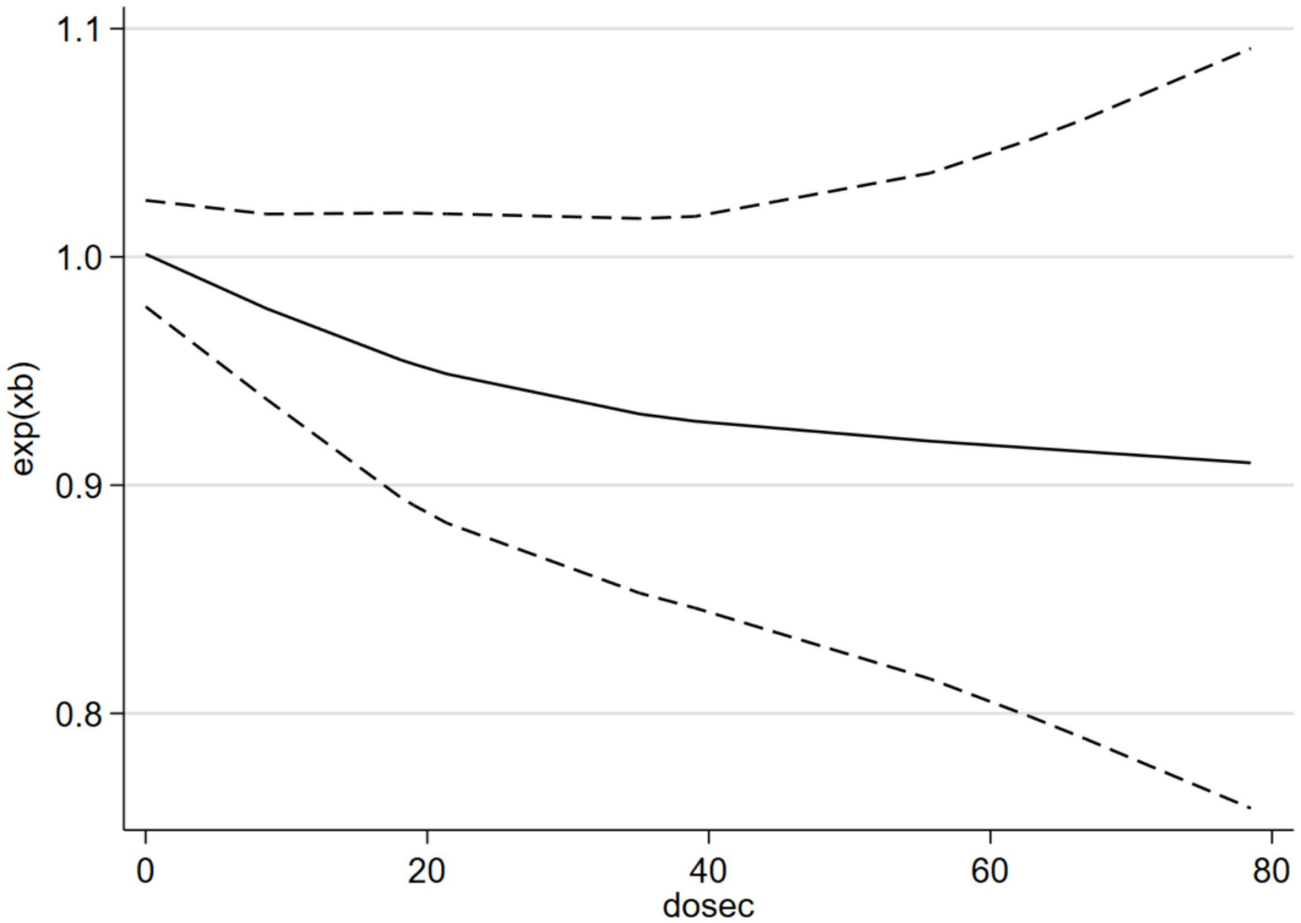
The dose–response relationship between i-MVPA and the risk of depression.

Figure 7 shows a positive non-linear response relationship between SB and risk of depression (*p*_non-linearity_ = 0.0287). When SB was <4 h/day, the OR of depression increased by 2% for every additional hour of SB per day SB (*p* > 0.05; OR = 1.02, 95% CI 0.97–1.07). When SB was >4 h/day, the OR for depression increased by 5% for every additional hour of SB per day (*p* < 0.05; OR = 1.05, 95% CI 1.01–1.12). Figure 8 shows a negative linear relationship between MVPA and risk of depression (*p*_non-linearity_ = 0.3253). The OR of depression was reduced by 3% for each 10 min/day increase (*p* < 0.05, OR = 0.97; 95% CI 0.90–1.00).

## 4 Discussion

This review and meta-analysis showed that both higher levels of i-SB and lower levels of i-PA were associated with a higher risk of depression when the most sedentary or least active groups of participants were compared to the least sedentary or most active groups. The highest OR was observed for TPA. After additional PA adjustment, i-SB, with a lower OR, remained not significantly associated with a higher risk of depression. After additional SB adjustment, i-PA, with a higher OR, was not significantly associated with a higher risk of depression. Meta-regression analyses showed clear dose–response relationships between i-SB, i-PA, and depression risk.

### 4.1 Observational studies on the association between i-SB and i-PA and depression risk

The OR for the association between i-SB and Depression Risk in this study (1.05, 95% CI 0.92–1.17) was not significant, possibly because some of the included literature in the study was adjusted OR by PA. This value was lower than that in previous meta-analyses on the association between self-reported sedentary time and depression among children and adolescents [OR = 1.14, 95% CI 1.08–1.20 (Zhang et al., 2022); OR = 1.12, 95% CI 1.03–1.22 (Liu et al., 2016)]. The ORs in previous meta-analyses among adults or older people [1.25, 95% CI 1.16–1.35 (Zhai et al., 2015); 1.28, 95% CI 1.17–1.39 (Wang et al., 2019)] were also higher than the ORs in the present study. Additionally, the non-significant and relatively low value likely resulted in an insignificant odds ratio (OR) in the continuous dose–response relationship outcomes. In real life, a daily sedentary time of fewer than 4 h may not affect mental and physical health (2–4 h is a non-linear relationship, as shown in Figure 7). However, in the analysis where daily sedentary time exceeded 2 h, a significant OR was observed, confirming that the OR for depression increased by 5% for every additional hour of SB per day. Consistent with the previous meta-analysis by Zhou et al. (2023), which did not specifically include i-SB studies, is that the association between SB and depression is non-linear, and prolonged SB is associated with a higher risk of depression. In contrast, the dose–response relationship curve presented by Zhou et al. (2023) indicates an inflection point at 6–8 h/day of SB, whereas the current study observed an inflection at 2–4 h/day. The potential reason for this discrepancy may lie in the insufficient number of studies included in the current analysis regarding the continuous dose–response of SB, as well as the influence of the previously mentioned physical activity (PA) adjustment. Therefore, caution should be exercised when interpreting this result. Additionally, a previous study also taken 1–2 h/day screen time in adolescent as the inflection point for risk of depression (Liu et al., 2016). In summary, Future research requires more robust examinations of the original dose–response relationship between objectively measured SB and depression to yield more reliable findings.

Regarding the association between MVPA and depression Risk, the OR in the present study (OR = 0.89, 95% CI 0.79–1.00) was almost the same as two previous meta-analyses included self-reported PA [OR = 0.90, 95% CI = 0.83–0.98 (Schuch et al., 2018); OR = 0.83, 95% CI 0.78–0.98 (Guo et al., 2022)]. SB is often underestimated and PA is often overestimated when using self-reported measures (Ryan et al., 2018). This may have influenced the accuracy of the estimation of depression risk in previous meta-analyses and observational studies. Recently, Gianfredi et al. (2020) conducted a meta-analysis of the associations between objectively measured PA and incident and prevalent depression, including 37,408 participants, with the overall effect size of depression for the highest vs. the lowest level of PA. However, their meta-analysis included studies with a patient group with chronic obstructive pulmonary disease or cancer and middle-aged and older populations, and there was significant publication bias and heterogeneity. Our meta-analysis specifically targeted adolescent populations and did not include studies involving patient groups. The ORs in our study included objective measures of PA and SB to analyze depression risk and were more targeted and precise.

Heterogeneity is a key indicator when testing the results of meta-analyses. We used meta-regression and subgroup analyses to explain the heterogeneity of our results in two respects: heterogeneity between subgroups and within groups. First, overall between-subgroup heterogeneity appeared in the different study design groups (cohort and cross-sectional) included in the i-SB study but not in the i-PA study. This may be due to the relatively large effect size of i-SB and the limited number of included studies. At the same time, the significant intergroup heterogeneity was also reflected in the analysis with respect to region and adjusted confounding factors (age and BMI) for i-SB, indicating that these factors might have influenced the study results. Future research requires more related literature for an in-depth investigation. Within-group heterogeneity was observed in the relevant i-PA and i-SB subgroups, suggesting that these results should be interpreted cautiously. This heterogeneity may also explain the source of overall heterogeneity. In addition, the heterogeneity between the subgroups of the different research types was not significant and therefore could not explain the overall heterogeneity. This indicates that there was no significant difference in the effect size between cross-sectional and longitudinal studies on the relationship between i-PA and depression risk. Finally, in analyzing the adjusted demographic confounding factors included in the literature, the between-group heterogeneity of age, sex, and BMI was significant, indicating that these three factors may have affected the overall heterogeneity.

This study presented the effect sizes (ORs) after mutual adjustment for PA and SB. The main finding was that after adjusting for PA, the OR for the relationship between SB and depression risk decreased. The OR with no adjusted PA was different from the adjusted OR with adjusted PA. This suggests that PA may mitigate the impact of SB on the risk of depression. Additionally, the OR for the relationship between MVPA and depression risk before adjusting for SB was higher than the OR after adjustment. This finding indicates that SB may not affect the preventive effects of MVPA on the risk of depression. These findings are consistent with a previous observational study on depression risk among adolescents (de Faria et al., 2022) as well as with a meta-analysis of all-cause mortality risk (Rojer et al., 2020). Finally, the ORs for TPA and LPA in relation to depression risk decreased after adjusting for MVPA level. However, the differences in heterogeneity between the groups were not significant. Further research is needed to confirm whether adjusting for different PA intensities affects the preventive effects of PA on the risk of depression.

The present study primarily employed two objective instruments: accelerometers and pedometers, regardless of the specific devices used or where they were worn. The absence of standardization among various measuring devices and improper installation of measurement components may be the underlying reasons for the overall heterogeneity. This issue was also observed in previous studies by Gianfredi et al. (2020). A study conducted by Montoye et al. (2016) compared the accuracy of accelerometers positioned on hips, thighs, and wrists. The results indicated that wrist-worn devices exhibited greater sensitivity and specificity than hip-worn devices in assessing SB and LPA. The thigh-worn device exhibited the highest sensitivity and specificity. Hence, to facilitate the comparison of various studies, it is crucial to use standardized methodologies and ensure the calibration of different devices when evaluating i-SB and i-PA.

### 4.2 Possible mechanisms for the role of i-SB and i-PA in depression

A range of neurobiological, psychosocial, and behavioral mechanisms for the role of PA in mental health and depression have been previously proposed by Lubans et al. (2016). First, PA may positively affect the brain’s structure and function, which is now quantifiable owing to technological advancements (e.g., magnetic resonance imaging). For instance, a randomized controlled trial in children showed that a 9-month physical exercise intervention improved the structure and function of the brain networks related to cognitive function (Hillman et al., 2014). Second, evidence suggests a causal link between physical self-concept (i.e., perceived appearance, fitness, and competence) and mental health (e.g., global self-concept and self-esteem) (Lubans et al., 2016). Social support and autonomy are plausible psychosocial contributors to mental health among young people (Hinkley et al., 2014). Finally, a range of potential behavioral mechanisms might explain the effect of PA on mental health outcomes, including sleep duration, sleep efficiency, sleep onset latency, and reduced sleepiness (Lubans et al., 2016).

Although the underlying mechanisms responsible for the effects of SB on mental health of children and adolescents remain unclear, several hypotheses have been proposed. First, given that SB often occurs alone, it may elicit feelings of loneliness and, consequently, negatively impact mental health (Hoare et al., 2016). Second, cultural messages transmitted through the media may affect other behaviors related to mental health (e.g., eating disorders and aggressive behavior) (Primack et al., 2009). Third, excessive media exposure often occurs during the day or at night. During the day, the time spent on screen-based activities may replace the time spent participating in more productive and/or active activities, particularly PA and interpersonal communication (Ohannessian, 2009). Since nothing is known about them, studies focusing on their neurobiological mechanisms are of particular interest.

### 4.3 Clinical implications

Quantifying the association between i-SB and i-PA and depression, as well as comparing the effect sizes obtained for i-SB and i-PA, are required for optimal PA-related risk management in children and adolescents. Clarifying this association is essential, as international activity guidelines (150 min of moderate-intensity PA or 75 min of vigorous-intensity PA) increasingly make strong recommendations to limit sedentary time or screen use in adolescents (World Health Organization [WHO], 2024). Our results emphasize the potential protective effects of reducing SB and promoting PA on mental health. Adolescents who were less sedentary had fewer depressive symptoms, supporting the recommendation to limit sedentary time as part of mental health prevention strategies. In clinical practice, this suggests that interventions should not only promote PA but also actively reduce SB, such as excessive screen time. Behavioral counseling in clinical settings can integrate advice on limiting sedentary activities and finding enjoyable physical activities that adolescents can maintain.

Moreover, the findings of these studies collectively highlight the need for interventions tailored to sex differences. Hamer et al. (2020) found significant associations in girls but not in boys, suggesting that the type, intensity, and context of PA might play different roles in mental health outcomes for different sexes. Clinicians should consider sex-specific approaches when designing and recommending PA programs.

Finally, the potential bidirectional relationship between depressive symptoms and PA or SB noted in these studies suggests that monitoring and supporting mental health in adolescents can concurrently encourage healthier lifestyle choices. Structured PA programs integrated into treatment plans may benefit adolescents with depressive symptoms. These programs should be designed to be engaging and feasible considering the motivational challenges faced by adolescents with depression.

In conclusion, although promoting PA and reducing SB are essential components of a healthy lifestyle for adolescents, these strategies alone may not be sufficient to prevent depression. A multifaceted approach, including early intervention, sex-specific strategies, and integration with broader mental health support, is recommended. Clinicians should work collaboratively with schools, communities, and families to implement these comprehensive interventions and ensure they are accessible and sustainable for all adolescents. Further research is needed to explore the nuanced relationships between these behaviors and mental health and to identify the most effective ways to support adolescent mental well-being.

### 4.4 Strengths and limitations

To our knowledge, this is the first systematic review and dose–response meta-analysis of the association between instrumented measures of SB, PA, and depression among children and adolescents. The use of exclusively objective measures of PA and SB represents a strength of this review, as questionnaires may not capture unstructured PA or LPA among children and adolescents are susceptible to over-reporting PA and under-reporting SB (Ryan et al., 2018; Dyrstad et al., 2014). Another strength of the present study is the quantification of the actual association between SB and PA and depression using instrumented measures, including accelerometers and pedometers. The present analyses assumed a low risk of reverse causation owing to the inclusion of cohort studies with various follow-up durations.

However, the potential limitations of this study should be considered. The first limitation is the relatively small number of articles included in our meta-analysis and the fact that the performed analyses used study-specific effect sizes that were all highly dependent on the measurement settings and analysis, as a range of different devices were included. Although we included both cohort and cross-sectional studies to increase the number of studies with objective evaluations, the number of relevant studies on adolescent populations remained limited. However, it is important to acknowledge that objective measures of PA and SB are limited in their capacity to measure the mode or type of PA or SB, including resistance loading during activities, which presents a limitation. The lack of a uniform definition for the assessment of i-SB and i-PA complicates the comparison of different studies, leading to the use of various cutoff values, devices, wearing locations, wearing times, and processing algorithms. Previous systematic reviews (Rojer et al., 2020) found five and eight different cutoff values for assessing SB and MVPA, respectively, even when using the same device. Finally, the five included studies employed a cross-sectional design; therefore, they were limited by methodological weaknesses. The cross-sectional characteristics of these studies do not allow causal inferences to be made because relationships cannot be determined.

### 4.5 Future research

Subsequent studies should prioritize the development of standardized techniques for measuring i-SB and i-PA, including recommendations for determining cutoff values, devices used, ideal duration of wear, and utilization of processing algorithms. These aspects might aim to directly examine the impacts of i-SB and i-PA on health-related outcomes. These findings provide valuable insights into the management of lifestyle-related risks by addressing both PA and SB. Additional longitudinal and interventional studies are necessary to investigate the combined relationship between PA and SB and the risk of depression in children and adolescents.

## 5 Conclusion

Both i-SB and i-PA were significantly associated with the risk of depression in children and adolescents, which may become non-significant after mutual adjustment for i-PA and i-SB. These findings highlight the importance of promoting PA and reducing SB in future lifestyle guidelines for children and adolescents. The implications of these results could guide school-based interventions to enhance physical activity. Future research should utilize standardized measurement methodologies and up-to-date processing techniques to recommend specific physical activity levels and limits to sedentary behavior.

## Data Availability

The original contributions presented in the study are included in the article/Supplementary material, further inquiries can be directed to the corresponding author.
