# Supplementary material for: Instrumented measures of sedentary behavior and physical activity are associated with depression among children and adolescents: a systematic review and dose–response meta-analysis of observational studies
Source: Front Psychol. 2024 Oct 4;15:1465974. doi: 10.3389/fpsyg.2024.1465974 (PMC11486697; doi:10.3389/fpsyg.2024.1465974)

# Supplementary Material Table S1-9.

# Full search strategy (conducted on 15-05-2024)

## Table S.1 Search strategy in PubMed (2024 MAY 15th)

| **#** | Query | Results |
| --- | --- | --- |
| **#7** | #5 AND #6 | \| **345** \|  \| \| --- \| --- \| |
| **#6** | ((((((((accelerometry[MeSH Terms]) OR (accelerometer)) OR (Acceleromet)) OR (pedometer)) OR (Monitoring, Physiologic)) OR (Monitoring, Ambulatory)) OR (actigraphy)) OR (objectively measured)) OR (Instrumented measure) | **1,424,642** |
| **#5** | #3 AND #4 | **1,730** |
| **#4** | "Exercise"[MeSH Terms] OR ("Exercises"[Title/Abstract] OR "physical activity"[Title/Abstract] OR "activities physical"[Title/Abstract] OR "activity physical"[Title/Abstract] OR "physical activities"[Title/Abstract] OR "exercise physical"[Title/Abstract] OR "exercises physical"[Title/Abstract] OR "physical exercise"[Title/Abstract] OR "physical exercises"[Title/Abstract] OR "acute exercise"[Title/Abstract] OR "acute exercises"[Title/Abstract] OR "exercise acute"[Title/Abstract] OR "exercises acute"[Title/Abstract] OR "exercise isometric"[Title/Abstract] OR "exercises isometric"[Title/Abstract] OR "isometric exercises"[Title/Abstract] OR "isometric exercise"[Title/Abstract] OR "exercise aerobic"[Title/Abstract] OR "aerobic exercise"[Title/Abstract] OR "aerobic exercises"[Title/Abstract] OR "exercises aerobic"[Title/Abstract] OR "exercise training"[Title/Abstract] OR "exercise trainings"[Title/Abstract] OR "training exercise"[Title/Abstract] OR (("education"[MeSH Subheading] OR "education"[All Fields] OR "Training"[All Fields] OR "education"[MeSH Terms] OR "train"[All Fields] OR "train s"[All Fields] OR "trained"[All Fields] OR "training s"[All Fields] OR "Trainings"[All Fields] OR "trains"[All Fields]) AND "Exercise"[Title/Abstract])) OR ("sedentary behavior"[MeSH Terms] OR ("behavior sedentary"[Title/Abstract] OR "sedentary behaviors"[Title/Abstract] OR "sedentary lifestyle"[Title/Abstract] OR "lifestyle sedentary"[Title/Abstract] OR "physical inactivity"[Title/Abstract] OR "inactivity physical"[Title/Abstract] OR "lack of physical activity"[Title/Abstract] OR "sedentary time"[Title/Abstract] OR "sedentary times"[Title/Abstract] OR "time sedentary"[Title/Abstract])) | **458,463** |
| **#3** | #1 AND #2 | **66,683** |
| **#2** | "depressive disorder"[MeSH Terms] OR "Depression"[MeSH Terms] OR "depressive symptoms"[Title/Abstract] OR "depressive symptom"[Title/Abstract] OR "symptom depressive"[Title/Abstract] OR "emotional depression"[Title/Abstract] OR "depression emotional"[Title/Abstract] | **291,745** |
| **#1** | "Adolescent"[MeSH Terms] OR "Adolescents"[Title/Abstract] OR "Adolescence"[Title/Abstract] OR "Teens"[Title/Abstract] OR "Teen"[Title/Abstract] OR "Teenagers"[Title/Abstract] OR "Teenager"[Title/Abstract] OR "Youth"[Title/Abstract] OR "Youths"[Title/Abstract] OR "adolescents female"[Title/Abstract] OR "adolescent female"[Title/Abstract] OR "female adolescent"[Title/Abstract] OR "female adolescents"[Title/Abstract] OR "adolescents male"[Title/Abstract] OR "adolescent male"[Title/Abstract] OR "male adolescent"[Title/Abstract] OR "male adolescents"[Title/Abstract] OR "child"[MeSH Terms] OR "Children"[Title/Abstract] | **3,822,002** |
| *Table S.2 Search strategy in Embase.com (2024 MAY 15th)* | |  |
| # | Query | Results |
| #17 | #13 AND #16 | **159** |
| #16 | #14 OR #15 | **58,406** |
| #15 | 'pedometer'/exp OR 'pedometry'/exp OR pedomet*:ab,ti,kw | **5,249** |
| #14 | 'accelerometry'/exp OR 'accelerometer'/exp OR 'actimetry'/exp OR 'actigraph'/exp OR acceleromet*:ab,ti OR actigra*:ab,ti | 56,883 |
| #13 | #7 AND #12 | **3,989** |
| #12 | #10 OR #11 | **802,058** |
| #11 | 'sedentary lifestyle'/exp OR 'sedentary lifestyle' OR 'sitting'/exp OR 'sitting' OR 'physical inactivity'/exp OR 'physical inactivity' OR sedent*:ab,ti,kw OR sitting:ab,ti,kw OR ((physical NEXT/1 inactivit*):ab,ti,kw) | **122,491** |
| #10 | #8 or #9 | **736,913** |
| #9 | 'exercises':ab,ti OR 'physical activity':ab,ti OR 'activities, physical':ab,ti OR 'activity, physical':ab,ti OR 'physical activities':ab,ti OR 'exercise, physical':ab,ti OR 'exercises, physical':ab,ti OR 'physical exercise':ab,ti OR 'physical exercises':ab,ti OR 'acute exercise':ab,ti OR 'acute exercises':ab,ti OR 'exercise, acute':ab,ti OR 'exercises, acute':ab,ti OR 'exercise, isometric':ab,ti OR 'exercises, isometric':ab,ti OR 'isometric exercises':ab,ti OR 'isometric exercise':ab,ti OR 'exercise, aerobic':ab,ti OR 'aerobic exercise':ab,ti OR 'aerobic exercises':ab,ti OR 'exercises, aerobic':ab,ti OR 'exercise training':ab,ti OR 'exercise trainings':ab,ti OR 'training, exercise':ab,ti OR 'trainings, exercise':ab,ti | **337,686** |
| #8 | 'physical activity'/exp | **564,390** |
| #7 | #3 AND #6 | **104,683** |
| #6 | #5 0R #4 | **688,720** |
| #5 | 'depressive symptom':ab,ti OR 'symptom, depressive':ab,ti OR 'emotional depression':ab,ti OR 'depression, emotional':ab,ti OR 'depressive symptoms':ab,ti | **91,755** |
| #4 | 'depression'/exp | **680,258** |
| #3 | #1 OR #2 | **3,585,543** |
| #2 | 'adolescents':ab,ti OR 'adolescence':ab,ti OR 'teens':ab,ti OR 'teen':ab,ti OR 'teenagers':ab,ti OR 'teenager':ab,ti OR 'youth':ab,ti OR 'youths':ab,ti OR 'adolescents, female':ab,ti OR 'adolescent, female':ab,ti OR 'female adolescent':ab,ti OR 'female adolescents':ab,ti OR 'adolescents, male':ab,ti OR 'adolescent, male':ab,ti OR 'male adolescent':ab,ti OR 'male adolescents':ab,ti OR 'child':ab,ti OR 'children':ab,ti | **2,308,899** |
| #1 | 'adolescent'/exp | **1,984,942** |

## Table S.3 Search strategy in the Cochrane Library (2024 MAY 15th)

| **#** | **Query** | **Results** |
| --- | --- | --- |
| **#17** | #15 and #16 | 189 |
| **#16** | (accelerometry or accelerometer or acceleromet or pedometer or Monitoring, Physiologic or Monitoring, Ambulatory or actigraphy or objectively measured or Instrumented measures) (Word variations have been searched) | **248230** |
| **#15** | #14 and #7 | **618** |
| **#14** | #10 and #13 | **140575** |
| **#13** | #11 or #12 | **11943** |
| **#12** | (Behavior, Sedentary):ti,ab,kw or (Sedentary Behaviors):ti,ab,kw or (Sedentary Lifestyle):ti,ab,kw or (Lifestyle, Sedentary):ti,ab,kw or (Physical Inactivity):ti,ab,kw or (Inactivity, Physical):ti,ab,kw or (Lack of Physical Activity):ti,ab,kw or (Sedentary Time):ti,ab,kw or (Sedentary Times):ti,ab,kw or (Time, Sedentary):ti,ab,kw | 11943 |
| **#11** | MeSH descriptor: [Sedentary Behavior] explode all trees | 1846 |
| **#10** | #8 or #9 | 139263 |
| **#9** | (Physical Activity):ti,ab,kw or (Activities, Physical):ti,ab,kw or (Activity, Physical):ti,ab,kw or (Physical Activities):ti,ab,kw or (Exercise, Physical):ti,ab,kw or (Exercises, Physical):ti,ab,kw or (Physical Exercise):ti,ab,kw or (Physical Exercises):ti,ab,kw or (Acute Exercise):ti,ab,kw or (Acute Exercises):ti,ab,kw or (Exercise, Acute):ti,ab,kw or (Exercises, Acute):ti,ab,kw or (Exercise, Isometric):ti,ab,kw or (Exercises, Isometric):ti,ab,kw or (Isometric Exercises):ti,ab,kw or (Isometric Exercise):ti,ab,kw or (Exercise, Aerobic):ti,ab,kw or (Aerobic Exercise):ti,ab,kw or (Aerobic Exercises):ti,ab,kw or (Exercises, Aerobic):ti,ab,kw or (Exercise Training):ti,ab,kw or (Exercise Trainings):ti,ab,kw or (Training, Exercise):ti,ab,kw or (Trainings, Exercise):ti,ab,kw | **128483** |
| **#8** | MeSH descriptor: [Exercise] explode all trees | **38714** |
| **#7** | #3 and #6 | 7969 |
| **#6** | #4 or #5 | 39775 |
| **#5** | (Depressive Symptoms):ti,ab,kw or (Depressive Symptom):ti,ab,kw or (Symptom, Depressive):ti,ab,kw or (Emotional Depression):ti,ab,kw or (Depression, Emotional):ti,ab,kw | 28766 |
| **#4** | MeSH descriptor: [Depression] explode all trees | **18240** |
| **#3** | #1 or #2 | **302036** |
| **#2** | (Adolescents):ti,ab,kw or (Adolescence):ti,ab,kw or (Teens):ti,ab,kw or (Teen):ti,ab,kw or (Teenagers):ti,ab,kw or (Teenager):ti,ab,kw or (Youth):ti,ab,kw or (Youths):ti,ab,kw or (Adolescents, Female):ti,ab,kw or (Adolescent, Female):ti,ab,kw or (Female Adolescent):ti,ab,kw or (Female Adolescents):ti,ab,kw or (Adolescents, Male):ti,ab,kw or (Adolescent, Male):ti,ab,kw or (Male Adolescent):ti,ab,kw or (Male Adolescents):ti,ab,kw or (child):ti,ab,kw or (children):ti,ab,kw | **295908** |
| **#1** | MeSH descriptor: [Adolescent] this term only | 136839 |
| *Table S.4 Search strategy in Scopus (*2024 MAY 15th*)* | |  |
| # | Query | Results |
| **S5** | S1 AND S2 AND S3 AND S4 | 702 |
| **S4** | All field: "Accelerometry" or "Accelerometer" or "Acceleromet" or "Pedometer" or "Monitoring, Physiologic" or "Monitoring, Ambulatory" or "Actigraphy" or "objectively measured" or "Instrumented measures" | **161,579** |
| **S3** | Article title, Abstact, keywords: "Physical Activity" or "Activities, Physical" or "Activity, Physical" or "Physical Activities" or "Exercise, Physical" or "Exercises, Physical" or "Physical Exercise" or "Physical Exercises" or "Acute Exercise" or "Acute Exercises" or "Exercise, Acute" or "Exercises, Acute" or "Exercise, Isometric" or "Exercises, Isometric" or "Isometric Exercises" or "Isometric Exercise" or "Exercise, Aerobic" or "Aerobic Exercise" or "Aerobic Exercises" or "Exercises, Aerobic" or "Exercise Training" or "Exercise Trainings" or "Training, Exercise" or "Trainings, Exercise" or "Sedentary Behavior" or "Behavior, Sedentary" or "Sedentary Behaviors" or "Sedentary Lifestyle" or "Lifestyle, Sedentary" or "Physical Inactivity" or "Inactivity, Physical" or "Lack of Physical Activity" or "Sedentary Time" or "Sedentary Times" or "Time, Sedentary" | **402,155** |
| **S2** | Article title, Abstact, keywords: "Depression" or "Depressive Symptoms" or "Depressive Symptom" or "Symptom, Depressive" or "Emotional Depression" or "Depression, Emotional" | **944,273** |
| **S1** | Article title, Abstact, keywords: "Adolescents" or "Adolescence" or "Teens" or "Teen" or "Teenagers" or "Teenager" or "Youth" or "Youths" or "Adolescents, Female" or "Adolescent, Female" or "Female Adolescent" or "Female Adolescents" or "Adolescents, Male" or "Adolescent, Male" or "Male Adolescent" or "Male Adolescents" or "child" or "children" | **5,195,529** |
| *Table S.5 Search strategy in PsycINFO via EBSCO (*2024 MAY 15th*)* | |  |
| # Query | | Results |

| **S5** | S1 AND S2 AND S3 AND S4 | **156** |
| --- | --- | --- |
| **S4** | TX All Text (Accelerometry* or "Accelerometer*" or "Acceleromet*" or "Pedometer*" or "Monitoring, Physiologic" or "Monitoring, Ambulatory" or "Actigraphy*" or "objectively measured" or "Instrumented measures") | **69,937** |
| **S3** | TI (Physical Activity* or "Activities, Physical" or "Activity, Physical" or "Physical Activities*" or "Exercise, Physical" or "Exercises, Physical" or "Physical Exercise" or "Physical Exercises" or "Acute Exercise" or "Acute Exercises" or "Exercise, Acute" or "Exercises, Acute" or "Exercise, Isometric" or "Exercises, Isometric" or "Isometric Exercises" or "Isometric Exercise" or "Exercise, Aerobic" or "Aerobic Exercise" or "Aerobic Exercises" or "Exercises, Aerobic" or "Exercise Training" or "Exercise Trainings" or "Training, Exercise" or "Trainings, Exercise" or "Sedentary Behavior" or "Behavior, Sedentary" or Sedentary Behaviors* or "Sedentary Lifestyle" or "Lifestyle, Sedentary" or "Physical Inactivity" or "Inactivity, Physical" or "Lack of Physical Activity" or "Sedentary Time" or "Sedentary Times" or "Time, Sedentary"  ) OR AB (Physical Activity* or "Activities, Physical" or "Activity, Physical" or "Physical Activities*" or "Exercise, Physical" or "Exercises, Physical" or "Physical Exercise" or "Physical Exercises" or "Acute Exercise" or "Acute Exercises" or "Exercise, Acute" or "Exercises, Acute" or "Exercise, Isometric" or "Exercises, Isometric" or "Isometric Exercises" or "Isometric Exercise" or "Exercise, Aerobic" or "Aerobic Exercise" or "Aerobic Exercises" or "Exercises, Aerobic" or "Exercise Training" or "Exercise Trainings" or "Training, Exercise" or "Trainings, Exercise" or "Sedentary Behavior" or "Behavior, Sedentary" or Sedentary Behaviors* or "Sedentary Lifestyle" or "Lifestyle, Sedentary" or "Physical Inactivity" or "Inactivity, Physical" or "Lack of Physical Activity" or "Sedentary Time" or "Sedentary Times" or "Time, Sedentary"  ) | **154,841** |
| **S2** | TI (Depression* or "Depressive Symptoms" or "Depressive Symptom" or "Symptom, Depressive" or "Emotional Depression" or "Depression, Emotional" ) OR AB (Depression* or "Depressive Symptoms" or "Depressive Symptom" or "Symptom, Depressive" or "Emotional Depression" or "Depression, Emotional" ) | **453,366** |
| **S1** | TI (Adolescents* or "Adolescence*" or "Teens*" or "Teen*" or "Teenagers*" or "Teenager*" or "Youth*" or "Youths*" or "Adolescents, Female" or "Adolescent, Female" or "Female Adolescent" or "Female Adolescents" or "Adolescents, Male" or "Adolescent, Male" or "Male Adolescent" or "Male Adolescents" or child* or "children") OR AB (Adolescents* or "Adolescence*" or "Teens*" or "Teen*" or "Teenagers*" or "Teenager*" or "Youth*" or "Youths*" or "Adolescents, Female" or "Adolescent, Female" or "Female Adolescent" or "Female Adolescents" or "Adolescents, Male" or "Adolescent, Male" or "Male Adolescent" or "Male Adolescents" or child* or "children") | **2,255,979** |

*Table S.6 Search strategy in SPORTDiscus via EBSCO (*2024 MAY 15th*)*

| # Query | Results |
| --- | --- |

| **S5** | S1 AND S2 AND S3 AND S4 | **132** |
| --- | --- | --- |
| **S4** | TX All Text (Accelerometry* or "Accelerometer*" or "Acceleromet*" or "Pedometer*" or "Monitoring, Physiologic" or "Monitoring, Ambulatory" or "Actigraphy*" or "objectively measured" or "Instrumented measures") | **76,898** |
| **S3** | TI (Physical Activity* or "Activities, Physical" or "Activity, Physical" or "Physical Activities*" or "Exercise, Physical" or "Exercises, Physical" or "Physical Exercise" or "Physical Exercises" or "Acute Exercise" or "Acute Exercises" or "Exercise, Acute" or "Exercises, Acute" or "Exercise, Isometric" or "Exercises, Isometric" or "Isometric Exercises" or "Isometric Exercise" or "Exercise, Aerobic" or "Aerobic Exercise" or "Aerobic Exercises" or "Exercises, Aerobic" or "Exercise Training" or "Exercise Trainings" or "Training, Exercise" or "Trainings, Exercise" or "Sedentary Behavior" or "Behavior, Sedentary" or Sedentary Behaviors* or "Sedentary Lifestyle" or "Lifestyle, Sedentary" or "Physical Inactivity" or "Inactivity, Physical" or "Lack of Physical Activity" or "Sedentary Time" or "Sedentary Times" or "Time, Sedentary"  ) OR AB (Physical Activity* or "Activities, Physical" or "Activity, Physical" or "Physical Activities*" or "Exercise, Physical" or "Exercises, Physical" or "Physical Exercise" or "Physical Exercises" or "Acute Exercise" or "Acute Exercises" or "Exercise, Acute" or "Exercises, Acute" or "Exercise, Isometric" or "Exercises, Isometric" or "Isometric Exercises" or "Isometric Exercise" or "Exercise, Aerobic" or "Aerobic Exercise" or "Aerobic Exercises" or "Exercises, Aerobic" or "Exercise Training" or "Exercise Trainings" or "Training, Exercise" or "Trainings, Exercise" or "Sedentary Behavior" or "Behavior, Sedentary" or Sedentary Behaviors* or "Sedentary Lifestyle" or "Lifestyle, Sedentary" or "Physical Inactivity" or "Inactivity, Physical" or "Lack of Physical Activity" or "Sedentary Time" or "Sedentary Times" or "Time, Sedentary"  ) | **203,348** |
| **S2** | TI (Depression* or "Depressive Symptoms" or "Depressive Symptom" or "Symptom, Depressive" or "Emotional Depression" or "Depression, Emotional"  ) OR AB (Depression* or "Depressive Symptoms" or "Depressive Symptom" or "Symptom, Depressive" or "Emotional Depression" or "Depression, Emotional"  ) | **331,991** |
| **S1** | TI (Adolescents* or "Adolescence*" or "Teens*" or "Teen*" or "Teenagers*" or "Teenager*" or "Youth*" or "Youths*" or "Adolescents, Female" or "Adolescent, Female" or "Female Adolescent" or "Female Adolescents" or "Adolescents, Male" or "Adolescent, Male" or "Male Adolescent" or "Male Adolescents" or child* or "children") OR AB (Adolescents* or "Adolescence*" or "Teens*" or "Teen*" or "Teenagers*" or "Teenager*" or "Youth*" or "Youths*" or "Adolescents, Female" or "Adolescent, Female" or "Female Adolescent" or "Female Adolescents" or "Adolescents, Male" or "Adolescent, Male" or "Male Adolescent" or "Male Adolescents" or child* or "children") | **1,748,292** |

*Table S.7 provides an overview of the included cohort and the relationship between sedentary behavior, physical activity, and depression in adolescents*

| **Author(year)** | **SB behaviour** | | **Mean SB duration (min/day)** | **depression n (%)** | **Effect size:age** |
| --- | --- | --- | --- | --- | --- |
|  | **Reported as** | **Cut-off** |  |  |  |
| Kracht et al., 2023 | SB (h/d) | SB (<25 counts/15 s) | 10.0(1.2) | 205 (11.7 %) | 1.01(0.99,1.02) |
| Hamer et al., 2020 | SB (min/day) | SB<100 cpm; | 391±46 | 6675(6.7 %) | 1.15(0.85,1.54) |
| Kandola et al., 2020 | SB (per 60 min) | SB: ≤199cpm; | 430±99min/day [65±80] | 3683(9.3%) | 1.24(1.08,1.45) |
| Slykerman et al., 2020 | SB (hours/week) | SB:<3METs=Actigraph count of 0-1135; | S1: Hours<10.5(75);  S2: Hours 10.5-12.0(242);  S3: >12.0 (86) | S1:10 (17.2);  S2:33 (56.9);  S3:15 (25.9) | 1.37(0.53,3.67) |
| da Costa et al., 2022 | SB (h/d） | SB: ≤35.6 mg | 10.25±1.34 | 610 (48.2%) | 0.9（0.79，1.03） |
| **Author(year)** | **LPA behaviour** | | **Mean LPA duration (min/day)** | **depression n (%)** | **Effect size:age** |
|  | **Reported as** | **Cut-off** |  |  |  |
| Hamer et al., 2020 | LPA (min/day) | LPA:100-2241cpm; | 284±37 | 6675(16.4 %) | 0.79(0.61,1) |
| Kandola et al., 2020 | LPA (per 60 min) | LPA:200-3599cpm; | 244±94min/day [SD55±08]) | 3683(9.3%) | 0.84(0.66,0.99) |
| Hagemann et al., 2021 | LPA（minutes) | n/r | Q1=218.57; Q2=252.42; Q3=292.16 | 470(51.06%) | 0.99(0.98,1) |
| da Costa et al., 2022 | LPA (h/day) | LPA:35.6 mg —201.4 mg | 4.21±0.98 | 610 (48.2%) | 0.90(0.85,1.18) |
| **Author(year)** | **MVPA behaviour** | | **Mean MVPA duration (min/day)** | **depression n (%)** | **Effect size:age** |
|  | **Reported as** | **Cut-off** |  |  |  |
| Kracht et al., 2023 | MVPA (min/d) | MVPA (≥574 counts/15 s) | 29.8(20.6,42.8) | 205 (11.7 %) | 0.44(0.02,0.87) |
| Booth et al., 2023 | MVPA (cpm) | 3600 counts-per-minute (cpm) | Males:29 minutes (SD = 17);  Females:18 minutes (SD=12) | S1:4755(Males=1784;  Females=2201) S2:4755(Males=1744; Females=2164) | 0.94(0.83,1.05) |
| Hamer et al., 2020 | MVPA (min/d) | MVPA:>2241.16cpm | 60±9 | 6675(6.7 %) | 0.85(0.58,1.25) |
| Kandola et al., 2020 | MVPA (per 15 mins) | MVPA: ≥3600cpm; | 325±66 min/day [SD58±09]) | 3683(9.3%) | 0.7(0.53,0.95) |
| Slykerman et al., 2020 | MVPA (hours/week) | MVPA:3-5.9METs=Actigraph count of 1136-3908 | S1: Hours <1.5(143);  S2: Hours 1.5-2.0(148);  S3:>2.0 (112) | S1:28 (48.3);  S2:16 (27.6);  S3:14 (24.1) | 0.64(0.35,1.16) |
| Toseeb et al., 2014 | MVPA ( min/d) | >4 METs | weekday: T1:<25.68, T2:25.68-72.79,  T3≥72.80 min/d;  weekend:T1:<15.73,T2:15.73-42.05,  T3:≥42.06 min/d | S1:718(3.37%); S2:671(4.17%) | 0.94(0.25,2.5) |
| Wiles et al., 2011 | MVPA (min/d) | Low:200-3,599cpm Medium:3,600-6,199cpm High:≥6,200cpm | 21 min/d | 2951(n/r) | 0.91(0.77,1.09) |
| Hume et al., 2011 | MVPA (mins/day) | n/r | S1:2004: Boys=105.3(±45.53);Girls=71.1(±27.99); S2:2006: Boys=55.9(±21.80);Girls=39.2(±19.04) | S1:2004:155(36.77%); S2:2006:155(43.03%) | 0.91(0.71,1.17) |
| Cushing et al., 2018 | MVPA(60min/day) | MVPA:≥9805 | 30.63±28.75 min | 26(4.28%) | 0.88(0.78,2.15) |
| da Costa et al., 2022 | MVPA (min/day) | MVPA:≥201.4 mg | 31.30±17.36 | 610 (48.2%) | 0.99(0.83,1.18) |
| Bell et al., 2019 | MPA | MPA:0.04≤AEE<0.10 kcal/kg/min | 14.95±1.36 h/d or 896.82 ± 81.69 min/day (17%) | 249(31 %) | 0.47(0.14,1.53) |
| **Author(year)** | **VPA behaviour** | | **Mean VPA duration (min/day)** | **depression n (%)** | **Effect size:age** |
|  | **Reported as** | **Cut-off** |  |  |  |
| Slykerman et al., 2020 | VPA( minutes/week) | VPA:≥6METs=Actigraph count of 3909-20000 | S1:Minutes <5;  S2:Minutes 5-15;  S3:Minutes >15 | S1:15(25.9); S2:28(48.3); S3:15 (25.9) | 1.1(0.46,2.6) |
| Clare Hume et al., 2011 | VPA (mins/day) | n/r | S1:2004:Boys=22.1(±37.04);Girls=10.9(±10.97); S2:2006:Boys=7.2 (±8.01);Girls=3.5 (±4.16) | S1:2004:155(36.77%); S2:2006:155(43.03%) | 0.87(0.44,1.75) |
| **Author(year)** | **TPA behaviour** | | **Mean TPA duration (min/day)** | **depression n (%)** | **Effect size:age** |
|  | **Reported as** | **Cut-off** |  |  |  |
| Wiles et al., 2011 | TPA | Low:200-3,599cpm Medium:3,600-6,199cpm High:≥6,200cpm | 21 min/d | 2951 (n/r) SMFQ | 0.7(0.58,0.85) |
| Hrafnkelsdottir et al., 2018 | TPA | TPA=1975cpm/day | 4 x/week | 244 (10.7%) | 1.24(0.6,2.56) |

SB:sedentary behaviour,LPA:light physical activity,MVPA moderate-to-vigorous physical activity,VPA:vigorous physical activity;TPA:total physical activity,PAEE:physical activity energy expenditure,METs:Metabolic Equivalents of Task,cpm：counts per minute, h/d:hours per day, min/day:minutes per day,SD:standard deviation,n/a:not applicable,n/r: not reported,Q=Quartile,T=tertiles,S=Subgroup,IQR:Interquartile Range,M:in male,F:in female.CAN:Canada,USA:United States of America,UK:United Kingdom,AUS:Australia,NZ:New Zealand,IS:Iceland,BR:Brazil.BE: Belgium

| *Table S.8. Characteristics of measurement methods used to assess instrumented sedentary behaviour and physical activity.* | | | | | | | |
| --- | --- | --- | --- | --- | --- | --- | --- |
| **Author** | **A/P** | **Device** | **Physical activity measures** | | **Assessment of valid days** | | **Mean wearing duration  (min/day)** |
|  |  |  | **Reported as** | **Cut-off value** | **Hours/day** | **Valid days** |  |
| **cohort studies** | | | | | | | |
| Kracht et al., 2023 | A | ActiGraphGT3X+ | SB; MVPA | SB (<25 counts/15 s); MVPA (≥574 counts/15 s) | ≥10 hours for≥4 days | 7 | 14.3 [1.2]h/d |
| Booth et al., 2023 | A | Actigraph AM 7164 | MVPA; TPA | 3600 counts-per-minute (cpm) | ≥3 days and 10 h of wear time per day | 6 | n/r |
| Hamer et al., 2020 | A | Actigraph GT1M; GENEActiv | MVPA; LPA; SB | SB<100cpm;LPA:100-2241cpm;MVPA:>2241.16cpm | ≥2 days with ≥10 hours | 7 | n/r |
| Kandola et al., 2020 | A | Actigraph 7164 or 71256 | SB; LPA; MVPA | SB: ≤199cpm; LPA:200-3599cpm; MVPA: ≥3600cpm; | >10 hours for≥3 days | 7 | n/r |
| Slykerman et al., 2020 | A | Actigraph (AM71256) | SB; MVPA; VPA | SB:<3METs=Actigraph count of 0-1135; MVPA:3-5.9METs=Actigraph count of 1136-3908; VPA:≥6METs=Actigraph count of 3909-20000 | 24 hours/day | 7 | 24 hours/day |
| Toseeb et al., 2014 | A | Actiheart,CamNtech Ltd | PAEE; MVPA | >4 METs | Weekend:43.10h/d(SD 6.47); Working day:60.75h/d(SD 12.41) | 6 | 24 hours/day |
| Hagemann et al., 2021 | A | Fitbit Charge 2 | LPA; MVPA | n/r | 24 hours/day,at least 3 days | 7 | 24 hours/day |
| Wiles et al., 2011 | A | Actigraph AM7164 | TPA; MVPA | Low:200-3,599cpm; Medium:3,600-6,199cpm; High: ≥6,200cpm | ≥10 hours for≥3 days | 7 | n/r |
| McKercher et al., 2014 | P | Yamax Digiwalker SW - 200 | Steps | Male child:1.00(low),3.45(medium),10.00(high) h /week;Female child:0.90(low),3.00(medium),8.00(high) h /week; | ≥8 hours for≥4 days | 7 | 8 hours/d |
| Olive et al., 2016 | P and A | New Lifestyle Pedometers; Actigraph GT1M | Steps; LPA; MVPA | LPA:101–2296 cpm;MVPA: >2297 cpm | ≥10 hours for≥ 3days | 7 | n/r |
| Bell et al., 2019 | A | Koninklijke PhilipsElectronics N.V. | SB; LPA; MPA; VPA | SB:AEE<0.01kcal/kg/min;LPA:0.01≤AEE<0.04kcal/kg/min;MPA:0.04≤AEE<0.10kcal/kg/min;VPA:AEE≥0.10 kcal/kg/min | ≥13 hours for≥ 4 days | 5 | 14.95±1.36 h/d or 896.82±81.69 min/day |
| **cross-sectional studies** | | | | | | | |
| Clare Hume et al., 2011 | A | Actigraph Model AM7164-2.2C | MVPA; VPA; SB | ≤50 counts/min; SB: METs≥3 | ≥6 hours for≥ 4 days | 7 | n/r |
| Cushing et al., 2018 | A | ActigraphLLC,Pensacola,FL | MVPA | sedentary:0-3660; light:3661-9804; MVPA: ≥9805 | 24 hours/day | 20 | 24 hours/day |
| da Costa et al., 2022 | A | ActiGraph GT3X+ | MVPA; LPA; SB | MVPA: ≥201.4 mg;LPA:35.6 mg—201.4 mg SB: ≤35.6 mg | ≥16 hours for ≥ 4 days | 7 | 24 hours/day |
| Farren et al., 2018 | A | ActiGraph GT1M | MVPA | n/r | ≥8 hours for ≥ 3 days | 7 | n/r |
| Hrafnkelsdottir et al., 2018 | A | Actigraph GT3X+ | VPA;TPA;screen time | total screen time:5.3 h/day; total PA =1975 cpm/day | ≥14 hours for ≥ 4 days | 7 | 5.3 h/day |

| *Table S.9. NOS Literature Quality Evaluation Table* | | | | | | | | | | | | | | | | | | | | | | |
| --- | --- | --- | --- | --- | --- | --- | --- | --- | --- | --- | --- | --- | --- | --- | --- | --- | --- | --- | --- | --- | --- | --- |
| cross-sectional studies | Selection | | | | | | | | | | | | | Comparability | | Outcome | | | | | | Quality score（10） |
| Author (year) | Representativeness of the sample（*） | | | | Sample size（*） | | | Non-respondents（*） | | | Ascertainment of the exposure (risk factor)（**） | | | Comparability and control of confounding factors（**） | | Assessment of outcome（**） | | | | Statistical test（*） | |  |
|  | a | b | c | d | a | b | c | a | b | c | a | b | c | a | b | a | b | c | d | a | b |  |
| Clare Hume et al., 2011 | * |  |  |  | * |  |  | * |  |  | ** |  |  | - |  |  | ** |  |  | * |  | 8 |
| Cushing et al., 2018 | * |  |  |  | - |  |  | - |  |  | ** |  |  | ** |  |  | ** |  |  | * |  | 8 |
| da Costa et al., 2022 | * |  |  |  | * |  |  | * |  |  | ** |  |  | ** |  |  | ** |  |  | * |  | 10 |
| Farren et al., 2018 | * |  |  |  | * |  |  | * |  |  | ** |  |  | ** |  |  | ** |  |  | * |  | 10 |
| Hrafnkelsdottir et al., 2018 | * |  |  |  | * |  |  | * |  |  | ** |  |  | ** |  |  | ** |  |  | * |  | 10 |
| cohort study | Selection | | | | | | | | | | | | | Comparability | | Outcome | | | | | | Quality score（9） |
| Author (year) | Representativeness of the exposed cohort (adult mixed gender or male or female)（*） | | | | Selection of the non-exposed cohort（*） | | | Ascertainment of exposure（*） | | | Demonstration that outcome of interest was not present at start of study（*） | | | Comparability of cohorts on the basis of the design or analysis（**） | | Assessment of outcome（*） | | Was follow-up long enough for outcomes to occur（*） | | Adequacy of follow up of（*） | |  |
| Kracht et al., 2023 | * | | | | * | | | * | | | * | | | ** | | * | | * | | * | | 9 |
| Booth et al., 2023 | * | | | | * | | | * | | | * | | | ** | | * | | * | | - | | 8 |
| Hamer et al., 2020 | * | | | | * | | | * | | | * | | | * | | * | | * | | - | | 7 |
| Kandola et al., 2020 | * | | | | * | | | * | | | * | | | * | | * | | * | | - | | 7 |
| Slykerman et al., 2020 | * | | | | * | | | * | | | * | | | - | | * | | * | | - | | 6 |
| Toseeb et al., 2014 | * | | | | * | | | * | | | * | | | ** | | * | | * | | - | | 8 |
| Hagemann et al., 2021 | * | | | | * | | | * | | | * | | | - | | * | | - | | - | | 5 |
| Wiles et al., 2011 | * | | | | * | | | * | | | * | | | ** | | * | | - | | - | | 7 |
| McKercher et al., 2014 | * | | | | * | | | * | | | * | | | ** | | * | | * | | * | | 9 |
| Olive et al., 2016 | * | | | | * | | | * | | | * | | | ** | | * | | * | | * | | 9 |
| Bell et al., 2019 | * | | | | * | | | * | | | - | | | ** | | * | | - | | - | | 6 |

Supplementary Figure 1A


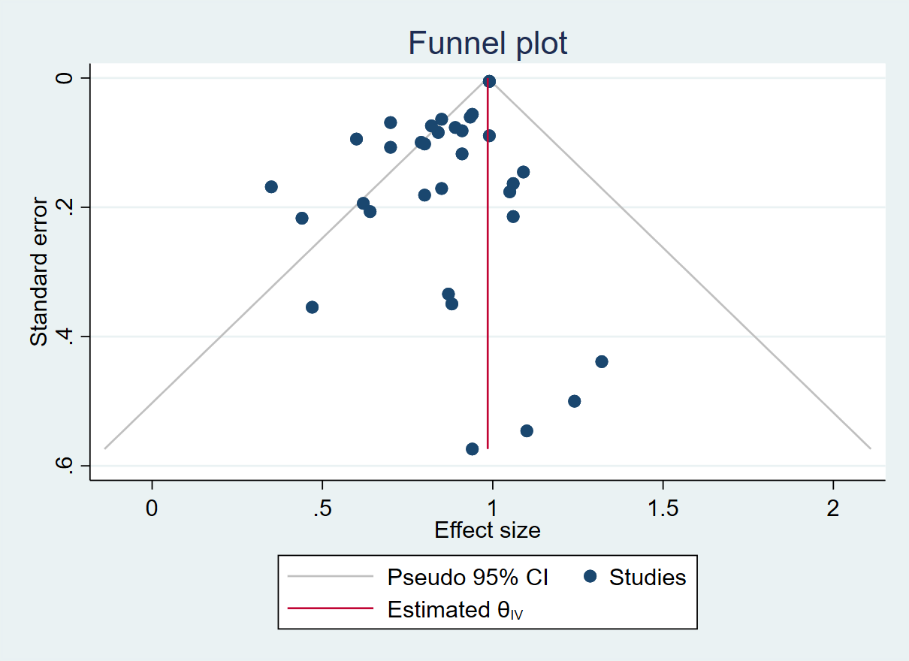


Supplementary Figure 1B


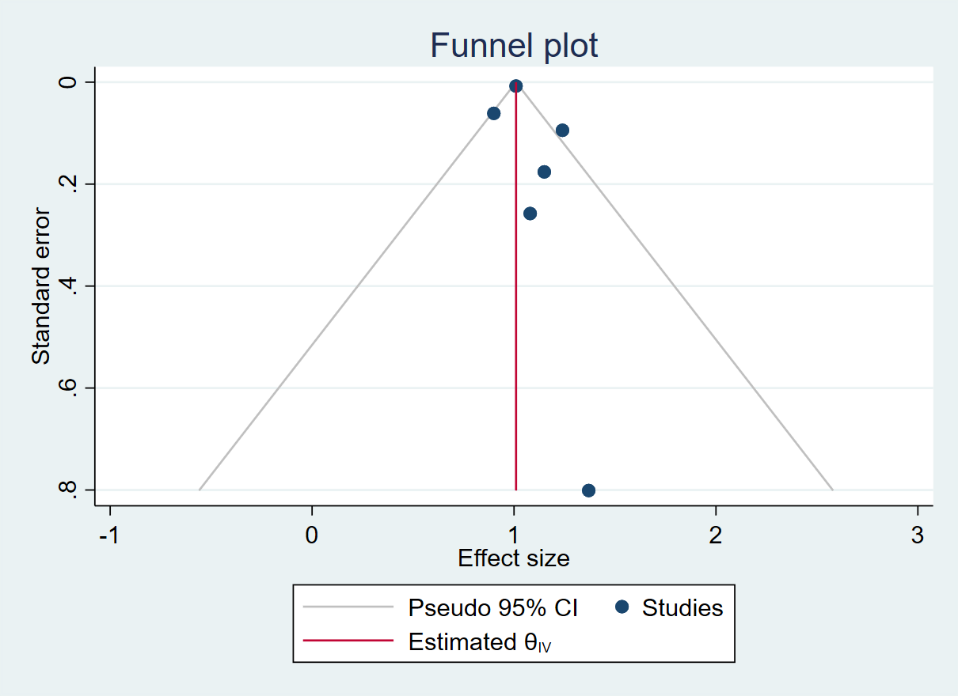

Supplement: Supplementary file 1 [file Table_1.DOCX]
